# PP2Acα regulates cerebellar development via phosphorylation-dependent neuronal programs

**DOI:** 10.1016/j.isci.2025.114352

**Published:** 2025-12-05

**Authors:** Yifan Li, Jing Ding, Simeng Liu, Qiao Wu, Yujie Fu, Qing Li, An Lv, Chunying Liu, Wei-Min Tong, Yamei Niu

**Affiliations:** 1 Department of Pathology, Institute of Basic Medical Sciences Chinese Academy of Medical Sciences, School of Basic Medicine Peking Union Medical College, Beijing 100005, China; 2 Center for Experimental Animal Research, Institute of Basic Medical Sciences Chinese Academy of Medical Sciences, Beijing 100005, China; 3 Molecular Pathology Research Center, Chinese Academy of Medical Sciences and Peking Union Medical College, Beijing 100005, China; 4 State Key Laboratory of Complex, Severe, and Rare Diseases, Peking Union Medical College Hospital, Chinese Academy of Medical Sciences and Peking Union Medical College, Beijing 100005, China

**Keywords:** Biochemistry, Neuroscience, Developmental biology

## Abstract

Cerebellar development requires precise control of phosphorylation, yet the contribution of protein phosphatases remains poorly understood. Here, we investigated the function of protein phosphatase 2A catalytic subunit alpha (PP2Acα) in cerebellar granule neurons. Conditional deletion of *Ppp2ca* using *Atoh1-Cre* mice resulted in impaired motor coordination and cognitive performance, reflecting disrupted cerebellar development. Histological analyses revealed abnormal granule neuron proliferation, migration, and defective Purkinje cell organization during early postnatal stages. Phosphoproteomic and proteomic profiling of primary granule neurons identified the PP2Acα-dependent regulation of cell cycle, cytoskeletal remodeling, RNA splicing, metabolism, and translation. Complementary profiling of whole cerebellar tissues uncovered broader system-level alterations affecting neuronal morphogenesis, mRNA processing, synaptic signaling, and behavior. Collectively, these findings establish PP2Acα as a critical regulator of cerebellar development through coordinated control of phosphorylation and protein homeostasis across cellular and system levels, providing mechanistic insights into phosphatase-related neurodevelopmental disorders.

## Highlights

• PP2Acα loss in granule neurons impairs motor coordination and behavior
• PP2Acα controls granule neuron proliferation, migration, and Purkinje cell organization
• Phosphoproteomics shows PP2Acα regulates cell cycle, RNA splicing, and cytoskeleton
• PP2Acα coordinates phosphorylation and proteostasis across cellular and system-levels

## Graphical abstract

**Figure:**
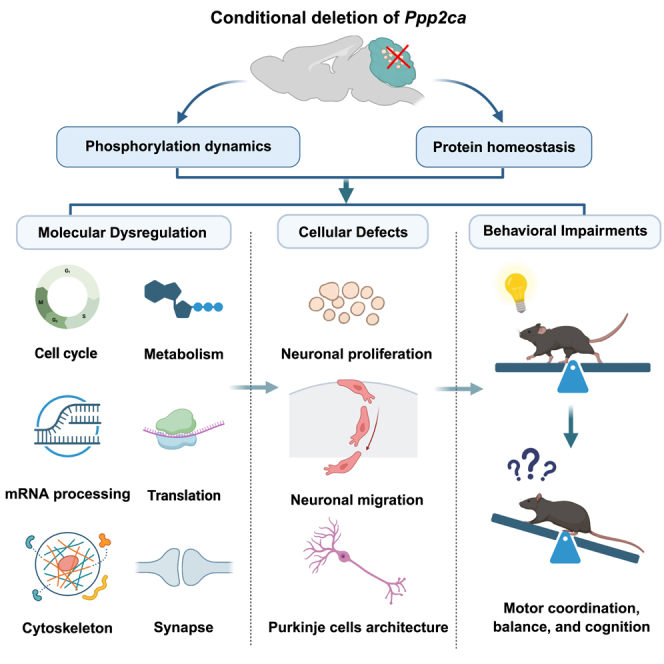

## Introduction

The cerebellum is a key brain region indispensable for the coordination of voluntary movement, balance, and higher-order cognitive function.^1^ Its intricate development involves a series of precisely regulated processes such as the proliferation and migration of neuronal precursors, differentiation of distinct neuronal subtypes, and formation of highly organized cerebral cortical layers and synaptic networks.^2^^,^^3^ These developmental events are orchestrated by a complex interplay of genetic programs and signaling pathways, operating in a tightly controlled spatial and temporal manner.^4^^,^^5^ At the protein level, post-translational modifications—particularly phosphorylation—serve as key regulatory mechanisms, modulating protein activity, localization, stability, and interactions.^6^ Multiple protein kinases, such as CDK5, ERK1/2 and PKA have been implicated in regulating various aspects of cerebellar development.^7^^,^^8^^,^^9^ By contrast, the essential dephosphorylating counterparts—protein phosphatases—remain largely understudied, particularly in the context of cerebellar development. Nevertheless, several studies have demonstrated that specific phosphatases play critical roles in cerebellar function. For instance, the Ca^2+^/calmodulin-dependent protein phosphatase 2B (PP2B) regulates synaptic potentiation and motor learning in Purkinje cells, and its genetic deletion leads to impaired long-term potentiation (LTP) and motor performance.^10^ Similarly, pharmacological inhibition of protein phosphatase 2A (PP2A) and PP2B has revealed their contribution to parallel fiber-Purkinje cell LTP.^11^ These findings underscore that phosphatase-mediated signaling is indispensable for cerebellar synaptic plasticity and behavior.

PP2A is one of the most abundant serine/threonine phosphatases in the brain, regulating neuronal signaling through the dephosphorylation of diverse substrates.^12^ Its roles in the cerebral cortex and hippocampus—such as controlling axonal growth, synaptic transmission, and neuroprotection—have been well documented.^13^^,^^14^^,^^15^^,^^16^^,^^17^ By contrast, investigations on PP2A in the cerebellum are comparatively sparse and often indirect. Nevertheless, a few emerging studies have begun to reveal the importance of PP2A-mediated signaling in maintaining cerebellar circuit homeostasis. For instance, studies on spinocerebellar ataxia type 1 (SCA1) suggest a reciprocal relationship between PP2A and ataxin-1, whereby PP2A dephosphorylates ataxin-1^18^ and ataxin-1 modulates PP2A activity and holoenzyme composition in Purkinje cells.^19^ Beyond these molecular interactions, pharmacological blockade of PP2A has been shown to regulate AMPA receptor clustering and synaptic efficacy at granule cell-Purkinje cell synapses, with inhibition occluding the induction of cerebellar long-term depression (LTD).^20^ More recently, the enhancement of PP2A activity in Purkinje cells was shown to convert cerebellar LTD into LTP,^21^ further highlighting the role of PP2A in cerebellar circuit function. Importantly, several studies have provided evidence that PP2A participates in cerebellar development. For example, the pharmacological inhibition of PP2A in cerebellar granule neuron precursors (CGNPs) modulates S6 kinase activity and inhibits CGNP proliferation and differentiation.^22^ PP2A-dependent Tau dephosphorylation underlies ceramide-induced cytoskeletal disruption and impaired neurite outgrowth in cerebellar granule neurons.^23^ However, these studies are largely indirect, and the precise molecular mechanisms underlying PP2A’s developmental roles remain unclear.

Among the catalytic subunits of PP2A, PP2Acα represents the dominant isoform in most tissues^24^^,^^25^ and is highly expressed in the cerebellum relative to other PP2A subunits.^12^ Importantly, *de novo* mutations in the *PPP2CA* gene are associated with neurodevelopmental disorders and reduced cerebellar volume in patients,^26^ underscoring its clinical relevance. Given that granule neurons represent the most abundant neuronal population in the cerebellum, we aimed to elucidate the specific functions of PP2Acα in these cells by generating a conditional *Ppp2ca* knockout mouse model. Using this model, we demonstrate that the granule neuron-specific deletion of PP2Acα impairs proliferation, migration, and connectivity, revealing that PP2Acα orchestrates diverse molecular programs—including cell cycle, metabolism, and cytoskeletal dynamics—essential for cerebellar development and function.

## Results

### Central nervous system-specific deletion of protein phosphatase 2A catalytic subunit alpha impairs cerebellar neuronal differentiation and maturation

**Figure 1:**
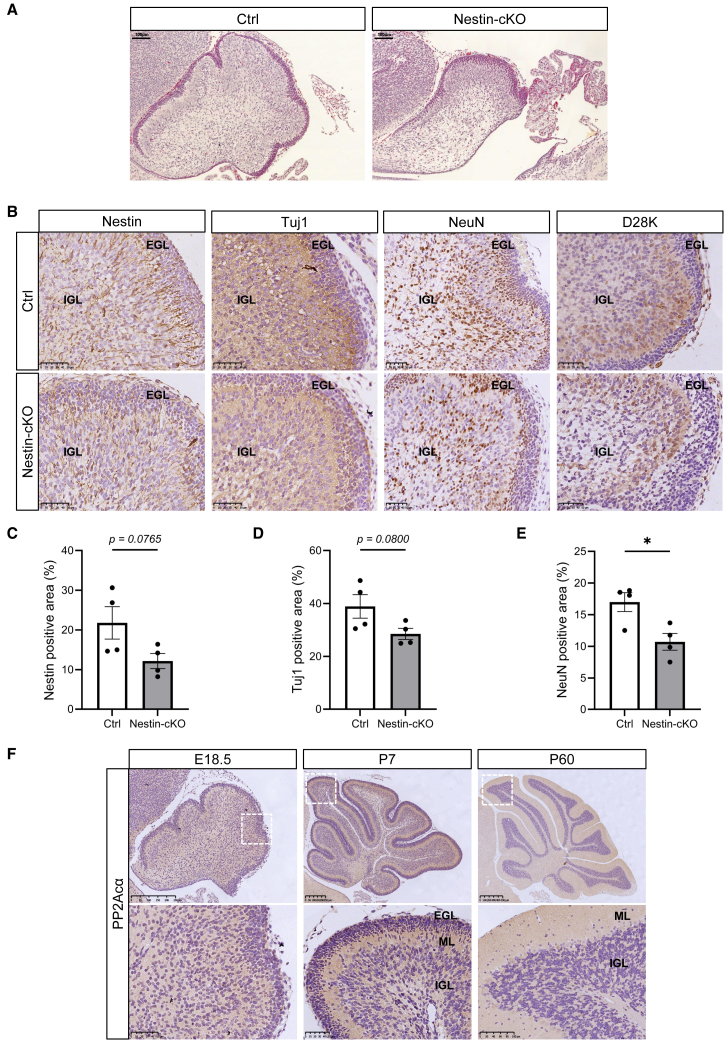
Conditional knockout of *Ppp2ca* in neural progenitors induces cerebellar structural and cellular alterations at E18.5 (A) Representative images of H&E staining of the cerebellum from Ctrl and Nestin-cKO mice. *n* = 4. Scale bars, 100 μm. (B) Immunohistochemical staining of Nestin, Tuj1, NeuN, and D28K in the cerebellum of Ctrl and Nestin-cKO mice. *n* = 4. Scale bars, 50 μm. (C–E) Comparison of Nestin- (C), Tuj1- (D), and NeuN- (E) positive area ratio in the cerebellum of Ctrl and Nestin-cKO mice. *n* = 4. Data are represented as mean ± SEM. ∗*p* < 0.05 (two-sided unpaired *t* test). (F) Immunohistochemical staining of PP2Acα in the cerebellum from the wild-type mice at different ages (E18.5, P7, and P60). Enlarged views of the boxed regions are shown in the lower panels. *n* = 3. Scale bars, 50 μm, 100 μm, 250 μm, and 500 μm. See also Figures S1 and S2.

We previously generated a central nervous system (CNS)-specific *Ppp2ca* knockout mouse (*Ppp2ca*^fl/fl^ *Nestin-Cre*^+^, hereafter referred to as Nestin-cKO), which exhibited severe defects in cerebral cortex neurogenesis.^14^ Given that *de novo PPP2CA* mutations are associated with reduced cerebellar volume in patients,^26^ we examined cerebellar morphology in Nestin-cKO mice to directly test its role in development. Compared with their littermate controls, Nestin-cKO mice at embryonic day 18.5 (E18.5) displayed significantly smaller cerebella with foliation defects (Figure 1A). Nestin immunostaining showed markedly reduced Nestin expression and disrupted radial fiber organization in both the external and the emerging internal granular layers (EGL and IGL) (Figures 1B, 1C, and S1A). Furthermore, Tuj1 immunostaining, a marker for immature and migrating neurons, showed overall reduced immunoreactivity and an accumulation of Tuj1-positive cells in the EGL, indicating impaired neuronal differentiation and delayed neuronal migration (Figures 1B, 1D, and S1A). Consistently, NeuN staining revealed fewer well-differentiated neurons in the IGL (Figures 1B, 1E, and S1A). Additionally, the continuity of Caibindin-D28K (D28K)-positive Purkinje cell layers was disrupted, with reduced staining intensity, suggesting compromised Purkinje cell development upon *Ppp2ca* deletion (Figures 1B and S1A).

To characterize the spatiotemporal and cell-type-specific expression of PP2Acα in the cerebellum, we next examined its mRNA and protein levels across developmental stages and different cell populations. RT-qPCR analysis revealed that *Ppp2ca* mRNA levels were highest at E18.5 and gradually decreased during postnatal development (Figure S1B). Consistently, PP2Acα protein expression showed a similar temporal pattern, with strong immunoreactivity at E18.5 and postnatal day 7 (P7) that markedly declined by P60 (Figure 1F). To further delineate its distribution across cerebellar cell types, we analyzed a published single-cell RNA-seq dataset from the mouse cerebellum.^27^ *Ppp2ca* transcripts were detected in nearly all major cerebellar cell populations, with particularly high levels in Purkinje cells and lower expression in granule neurons (Figure S1C). In line with these transcriptomic data, immunofluorescence staining revealed the broad expression of PP2Acα protein across major cerebellar types, from granule neurons and Purkinje cells to astrocytes (Figure S2). Moreover, PP2Acα was present throughout the neuronal differentiation process, as shown by co-immunostaining with stage-specific neuronal markers—Nestin (neural progenitors), Tuj1 (immature neurons), and NeuN (mature neurons), suggesting a role in multiple stages of cerebellar neuron development.

Together, these data demonstrate that PP2Acα is widely expressed in the cerebellum during embryonic and early postnatal stages, suggesting a potential role for neuronal differentiation and cerebellar morphogenesis.

### Granule neuron-specific deletion of protein phosphatase 2A catalytic subunit alpha causes cerebellar structural abnormalities and functional deficits

Given the severe cerebellar malformations observed in Nestin-cKO mice, we next investigated the role of PP2Acα in cerebellar granule neurons, the most abundant neuronal subtype in the cerebellum. To this end, we generated conditional knockout (Atoh1-cKO) mice by crossing *Ppp2ca*^fl/fl^ mice with *Atoh1*-*Cre* transgenic mice,^28^ which specifically target granule neuron progenitors (Figures S3A and S3B). Western blot (WB) and immunohistochemistry (IHC) analyses confirmed a robust reduction of PP2Acα protein in the cerebella of Atoh1-cKO mice (Figures S3C and S3D). These mice were viable, with observed genotypes consistent with Mendelian inheritance (Figure S3E), indicating that *Ppp2ca* deletion in granule neurons did not impair embryonic development or postnatal survival.

**Figure 2:**
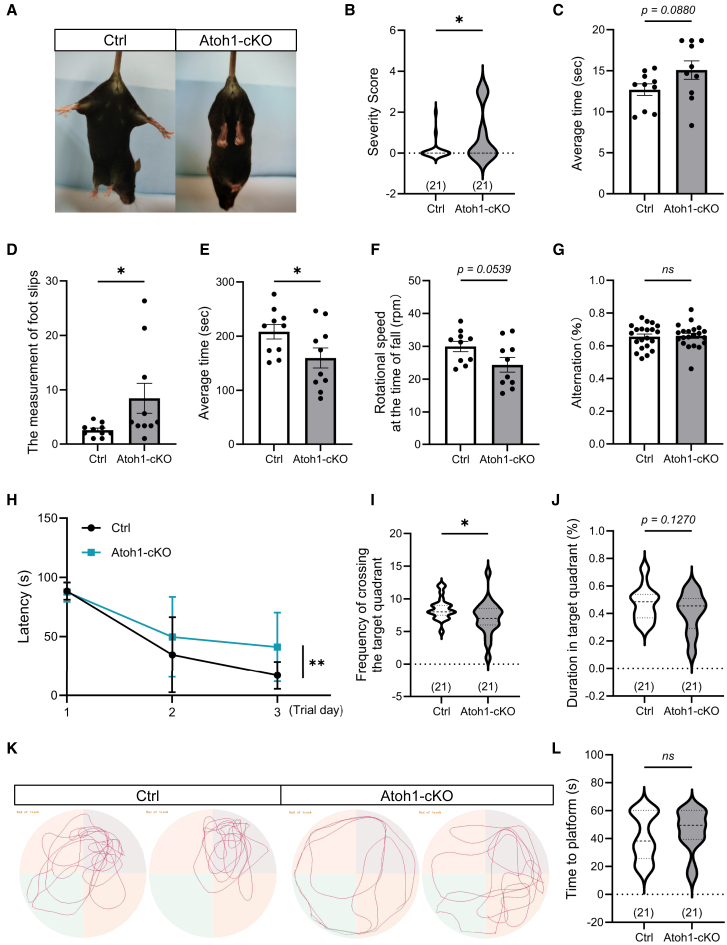
Conditional knockout of *Ppp2ca* in cerebellar granule neurons results in motor coordination and cognitive deficits in 3-month-old mice (A) Representative images show hindlimb clasping phenotype during tail suspension. (B) Quantification of hindlimb clasping scores in Ctrl and Atoh1-cKO mice. *n* = 21. (C and D) Latency to traverse the balance beam (C) and the measurement of foot slips (D). *n* = 10. (E and F) Latency to fall in the rotarod test (E) and the rotational speed at the time of fall (F). *n* = 10. (G) Spontaneous arm alternation in the Y maze test. *n* = 21. (H) Escape latency during training days in the Morris Water Maze. *n* = 21. (I and J) Probe trial analysis in the Morris Water Maze: frequency of target quadrant entries (I) and percentages of time spent in the target quadrant within 60 s (J). *n* = 21. (K) Representative images showing path tracing in the Morris Water Maze. (L) Escape latency to reach the visible platform in the Morris Water Maze within 60 s *n* = 21. Data are represented as mean ± SEM. ∗*p* < 0.05, ∗∗*p* < 0.01 and *ns*: non-significant (two-sided unpaired *t* test, two-sided one-way ANOVA). See also Figures S3 and S4.

To evaluate neurological functions, we performed a series of behavioral tests in adult control (Ctrl) and Atoh1-cKO mice. Hindlimb-clasping, an indicator of neurological dysfunction,^29^ revealed that while Ctrl mice maintained normal limb extension when suspended (score 0–1), Atoh1-cKO mice exhibited a higher proportion of clasping involving both hind- and forelimbs (score 1–3) (Figures 2A and 2B). In the balance beam assay, Atoh1-cKO mice required more time to traverse a 1-m beam (Figure 2C) and displayed significantly more hindlimb slips compared with controls (Figure 2D). Similarly, in the accelerating rotarod test (5–40 rpm over 5 min), Atoh1-cKO mice fell earlier and at lower speeds than controls (Figures 2E and 2F), indicating impaired motor coordination and balance. These results demonstrate that the deletion of PP2Acα in granule neurons compromises cerebellum-dependent motor coordination and balance.

Given the cerebellum’s role in cognition,^30^ we further assessed cognitive performance using the Y-maze and Morris water maze. No differences were observed in the Y-maze test (Figure 2G). In the hidden platform phase of the Morris water maze, however, Atoh1-cKO mice displayed significantly prolonged escape latencies (Figure 2H). In the subsequent probe trial, they exhibited a reduced frequency of target quadrant entries (Figure 2I) and a lower percentage of time spent in the target quadrant (Figures 2J and 2K). During the visible platform phase, however, no differences were detected between groups, indicating that the observed deficits were not due to visual or basic motor impairments (Figure 2L).

To determine whether these behavioral phenotypes were associated with structural abnormalities, we examined the cerebella of adult mice (3 months old). While body weight was unaffected (Figure S4A), brain weight was significantly reduced (Figures S4B and S4C). Furthermore, Hematoxylin-eosin (H&E) staining revealed an overall smaller cerebellum and reduced cell density in the IGL (Figure S4D).

Together, these findings establish that PP2Acα in granule neurons is critical for proper cerebellar morphogenesis and acquisition of motor and cognitive functions.

### Granule neuron-specific deletion of protein phosphatase 2A catalytic subunit alpha disrupts proliferation, cell cycle progression, and migration during cerebellar development

**Figure 3:**
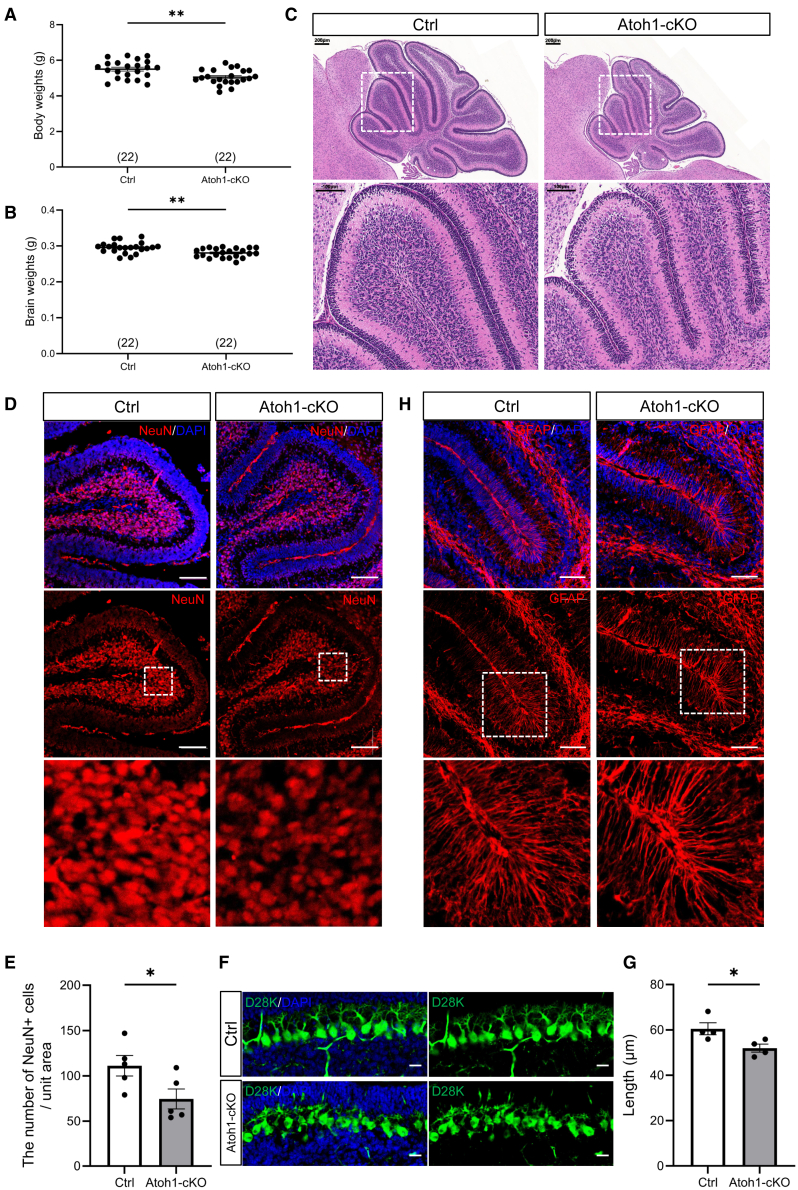
Granule neuron-specific PP2Acα deficiency selectively affects specific neuronal populations in the cerebellum of P8 mice (A and B) Quantitative comparison of body weights (A) and brain weights (B) of individual Ctrl and Atoh1-cKO mice. (C) H&E staining of cerebellar sections from Ctrl and Atoh1-cKO mice. *n* = 4. Scale bars, 100 μm and 200 μm. (D) Immunofluorescence staining of NeuN (Red) in the cerebellum of Ctrl and Atoh1-cKO mice. *n* = 5. Scale bars, 100 μm. (E) Comparison of NeuN-positive mature neuron density in the cerebellum of Ctrl and Atoh1-cKO mice. *n* = 5. (F) Immunofluorescence staining of Calbindin-D28K (Green)-positive Purkinje cells in the cerebellum of Ctrl and Atoh1-cKO mice at P8. *n* = 4. Scale bars, 25 μm. (G) Comparison of Purkinje neuron dendritic length in the cerebellum of Ctrl and Atoh1-cKO mice. *n* = 4. Data are represented as mean ± SEM. ∗*p* < 0.05 and ∗∗*p* < 0.01 (two-sided unpaired *t* test). (H) Immunofluorescence staining of GFAP (Red)-positive astrocytes in the cerebellum of Ctrl and Atoh1-cKO mice. *n* = 3. Scale bars, 100 μm. DAPI was used for the counterstaining of the nucleus. See also Figures S5 and S6.

Because the behavioral and structural deficits observed in adult Atoh1-cKO mice likely result from disrupted cerebellar development, we next examined early postnatal stages to investigate the underlying cellular mechanisms. P8 cerebella were primarily analyzed for granule neuron proliferation, migration, and layer formation, with P14 included to assess the persistence of any abnormalities. At P8, Atoh1-cKO mice displayed slightly reduced body and brain weights compared with littermate controls (Figures 3A, 3B, and S5A). H&E staining revealed a substantial reduction in cerebellar volume, although overall cerebellar morphology and lobule structure were grossly preserved (Figure 3C). Notably, the EGL appeared thinner, and the IGL showed markedly decreased cell density. Similar reductions in brain weight and cerebellar size were observed at P14 (Figures S5B–S5E), indicating persistent developmental deficits.

Furthermore, immunofluorescence analyses of P8 cerebella demonstrated a significant reduction in NeuN-positive mature neurons within the IGL of Atoh1-cKO mice (Figures 3D and 3E). While the number of Purkinje cells, identified by D28K staining, was not significantly altered, their dendritic architecture appeared disorganized and shorter (Figures 3F and 3G). In contrast, astrocytes labeled by glial fibrillary acid protein (GFAP) exhibited no noticeable changes in density or morphology (Figure 3H). IHC at both P8 (Figure S6A) and P14 (Figure S6B) confirmed reduced NeuN-positive neuron density and persistent Purkinje cell disorganization.

**Figure 4:**
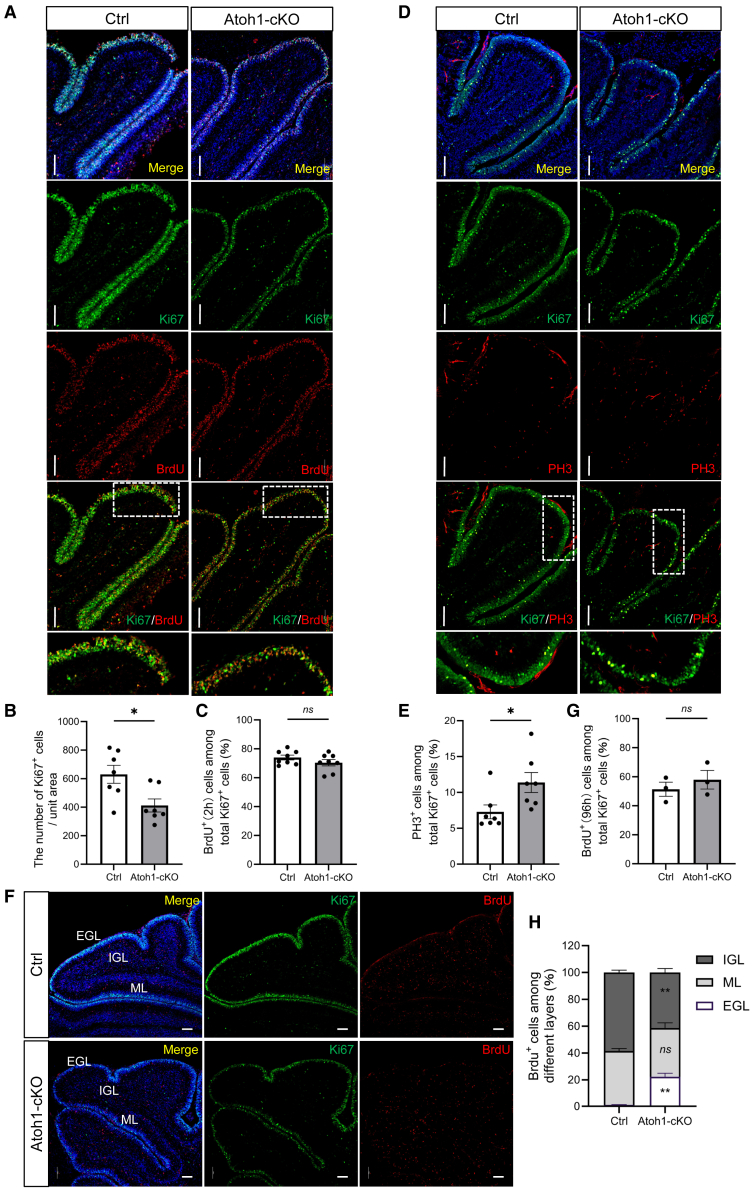
Granule neuron-specific PP2Acα deficiency impairs neuronal progenitor proliferation and migration in the cerebellum of P8 mice (A) Immunofluorescence staining of BrdU (2 h) and Ki67 in the cerebellum of Ctrl and Atoh1-cKO mice at P8. Scale bars, 100 μm. (B) Comparison of Ki67-positive cell density in the cerebellum Ctrl and Atoh1-cKO mice at P8. *n* = 7. (C) Proportion of BrdU^+^/Ki67^+^ cells among total Ki67^+^ cells. *n* = 4 biologically independent mice; for each mouse, data from 2 cerebellar lobules were pooled for analysis. (D) Immunofluorescence staining of Ki67 and PH3 in the cerebellum of Ctrl and Atoh1-cKO mice at P8. Scale bars, 100 μm. (E) Proportion of PH3^+^/Ki67^+^ cells among total Ki67^+^ cells. *n* = 3 biologically independent mice; for each mouse, data from 2 or 3 cerebellar lobules were pooled for analysis. (F) Immunofluorescence staining of BrdU (96 h) and Ki67 in the cerebella of Ctrl and Atoh1-cKO mice at P8. Scale bars, 100 μm. (G) Proportion of BrdU^+^ cells among total Ki67^+^ cells. *n* = 3. (H) Proportions of BrdU-labeled cells across the EGL, ML, and IGL. *n* = 3. DAPI was used for the counterstaining of the nucleus. Data are represented as mean ± SEM. ∗*p* < 0.05, ∗∗*p* < 0.01, and *ns*: non-significant (two-sided unpaired *t* test). See also Figure S6.

We next investigated whether the reduction of cerebellar neurons resulted from impaired granule cell proliferation. Immunofluorescence and IHC analyses showed a significant reduction of Ki67-labeled cells in the EGL of P8 Atoh1-cKO mice (Figures 4A, 4B, and S6C). Subsequently, 5-Bromo-2-deoxyUridine (BrdU) incorporation assays showed that, despite the overall reduction of Ki67-positive cells, the ratio of BrdU-positive to Ki67-positive cells remained comparable between groups, indicating that entry into S phase was not largely affected (Figures 4A and 4C). However, the ratio of phospho-histone H3 (PH3)-positive to Ki67-positive cells was significantly increased in the Atoh1-cKO mice, suggesting an accumulation of cells in M phase (Figures 4D, 4E, and S6C).

Given that proper proliferation and cell cycle progression are prerequisites for the radial migration of granule neuron progenitors from the EGL to the IGL, we next assessed granule neuronal migration using BrdU labeling at P4 and analyzed 96 h later. In P8 Atoh1-cKO mice, a higher proportion of BrdU-labeled cells remained in the EGL, while fewer had migrated into the IGL compared with the Ctrl mice (Figures 4F–4H).

Together, these findings demonstrate that PP2Acα in granule neurons is essential for cerebellar development, coordinating proliferation, cell cycle progression, and migration of granule neuron progenitors.

### Phosphoproteomic and proteomic analyses in primary granule neurons reveal the PP2Acα-dependent regulation of cell cycle, cytoskeletal organization, metabolism, and translation

To elucidate the molecular mechanisms underlying the cerebellar developmental defects in the Atoh1-cKO mice, we performed phosphoproteomic and global proteomic analyses in primary granule neurons isolated from Ctrl and Atoh1-cKO mice (Figure S7).

**Figure 5:**
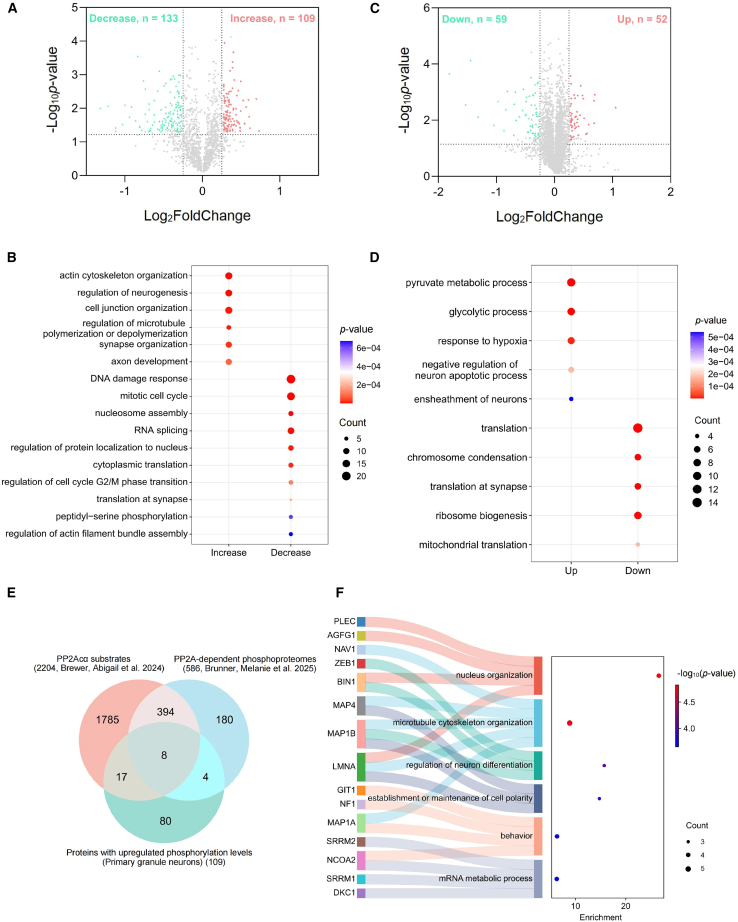
Granule neuron-specific PP2Acα deficiency induces widespread phosphorylation rewiring and proteomic alterations in primary granule neurons (A) Volcano plot shows differentially phosphorylated proteins. (B) GO functional enrichment analysis of the proteins with increased or decreased phosphorylation. (C) Volcano plot shows differentially expressed proteins. (D) GO functional enrichment analysis of up- or downregulated proteins. (E) Venn diagram shows the potential substrate proteins of PP2Acα in primary granule neurons. (F) Sankey diagram shows the GO functional classification of the candidate substrates of PP2Acα. See also Figures S7 and S8 and Tables S1, S2, and S3.

In the phosphoproteomic dataset, 35,636 high-confidence spectra were identified from a total of 319,487 total spectra (False discovery rate [FDR] ≤ 0.01). We detected 3,851 phosphorylated peptides and 3,034 phosphorylated sites (phosphoRS probability ≥0.75), mapping to 1,532 phosphoproteins (Figure S8A). Using thresholds of |FoldChange (cKO/Ctrl)| > 1.2 and *p*-value <0.05, we identified 109 proteins with increased phosphorylation and 133 proteins with decreased phosphorylation in Atoh1-cKO neurons (Figure 5A and Table S1). Gene Ontology (GO) enrichment analysis revealed that proteins with increased phosphorylation were mainly involved in neurodevelopmental and cytoskeletal processes, including actin cytoskeleton organization, regulation of neurogenesis, cell junction organization, regulation of microtubule polymerization/depolymerization, synapse organization, and axon development. Conversely, proteins with decreased phosphorylation were enriched in pathways related to DNA damage response, mitotic cell cycle, nucleosome assembly, RNA splicing, and cytoplasmic translation (Figure 5B). Notably, several affected proteins (BRCA1, CDK1, CDK2, PBIR1) are key regulators of G2/M-phase progression,^31^^,^^32^^,^^33^^,^^34^ consistent with the M-phase accumulation observed in PP2Acα-deficient granule neurons. In addition, the reduced phosphorylation of certain peptidyl-serine phosphatases suggested potential feedback disruptions within protein phosphatase signaling networks.

Global proteomic analysis identifies 29,123 unique peptide segments corresponding to 5,569 proteins from 365,413 spectra (FDR ≤0.01) (Figure S8B). Differential expression analysis (|FoldChange (cKO/Ctrl)| > 1.2, *p*-value <0.05) revealed 111 differentially expressed proteins, including 52 upregulated and 59 downregulated proteins (Figure 5C and Table S2). GO analysis showed that upregulated proteins were mainly involved in the pyruvate metabolic process, glycolytic process, and regulation of neuron apoptotic process, whereas downregulated proteins were enriched in translation-related pathways (Figure 5D), indicating suppressed ribosome biogenesis and reduced translational capacity.

To identify potential direct PP2Acα substrates, we intersected the proteins with increased phosphorylation in Atoh1-cKO neurons with two previously published PP2A-dependent phosphoproteomic datasets (2,204 PP2Acα substrates in HEK293 cells and 586 PP2A-dependent phosphoproteins in A549 and HeLa cells).^35^^,^^36^ This analysis yielded 29 candidate substrates in the cerebellum (Figure 5E and Table S3), which are implicated in nucleus organization, cytoskeleton organization, neuron differentiation, cell polarity, behavior, and mRNA metabolism (Figure 5F).

Collectively, these data indicate that PP2Acα regulates granule neuron development through phosphorylation-dependent pathways controlling cell cycle progression, cytoskeletal dynamics, and synaptic function, as well as metabolic and translational homeostasis.

### System-level proteomic profiling reveals the protein phosphatase 2A catalytic subunit alpha-dependent regulation of cerebellar structure and development

Although PP2Acα deletion was restricted to granule neurons using the *Atoh1-Cre* driver, cerebellar development and function depend on coordinated interactions among multiple cell types, including Purkinje cells and glia.^37^^,^^38^ To capture system-level alterations beyond cell-autonomous effects, we performed mass spectrometry-based phosphoproteomic and proteomic analyses on whole cerebellar tissue from Atoh1-cKO and Ctrl mice.

**Figure 6:**
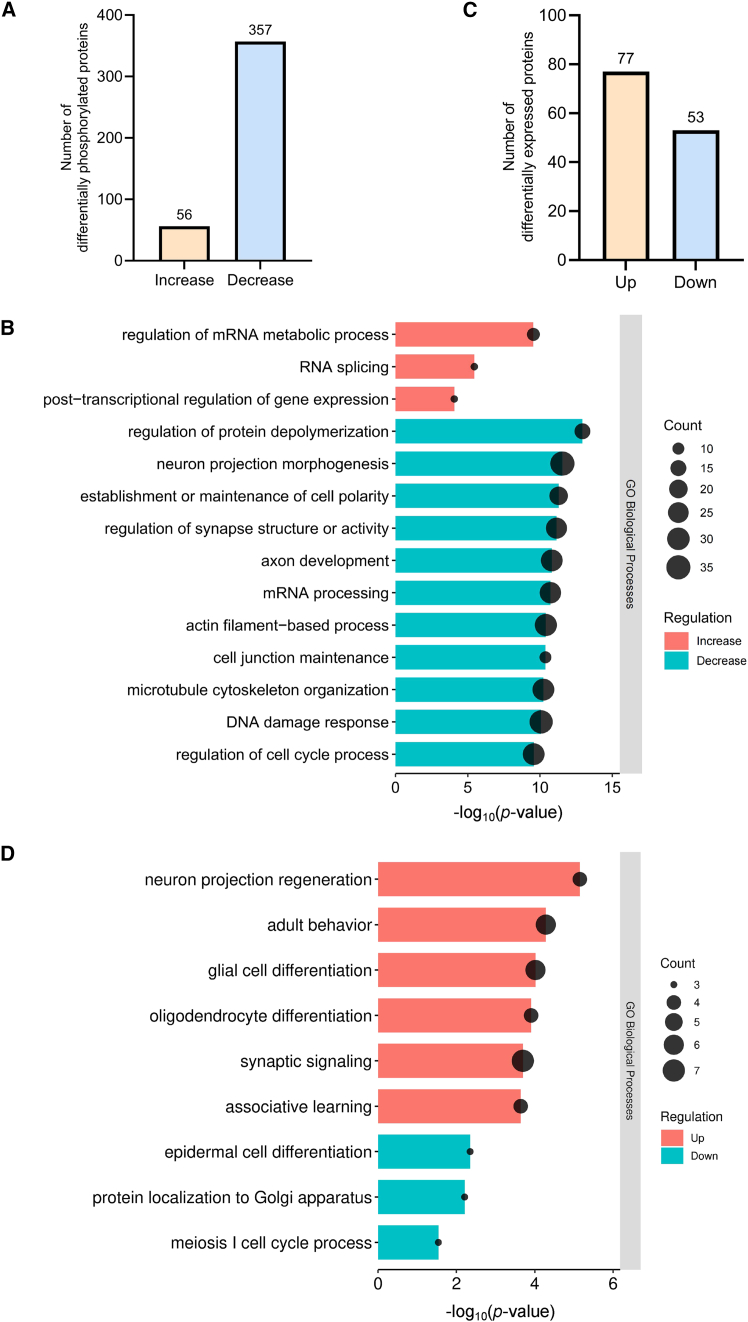
Phosphoproteomic and proteomic profiling revealed functional pathway alterations in the cerebellum of P8 Atoh1-cKO mice (A) Boxplot shows the numbers of differentially phosphorylated proteins. (B) GO functional enrichment analysis of proteins with increased or decreased phosphorylation in cerebellar tissue. (C) Boxplot shows the numbers of differentially expressed proteins. (D) GO functional enrichment analysis of up- and downregulated proteins in cerebellar tissue. See also Figure S9 and Tables S4 and S5.

Phosphoproteomic analysis yielded 36,766 high-confidence spectra corresponding to 12,145 peptides, 3,655 proteins, and 1,790 quantifiable phosphoproteins (Figure S9A). Compared with Ctrl mice, 56 proteins showed increased phosphorylation, whereas 357 proteins exhibited decreased phosphorylation in Atoh1-cKO mice (|FoldChange (cKO/Ctrl)| > 1.2, *p*-value <0.05) (Figure 6A and Table S4). GO enrichment analysis indicated that proteins with decreased phosphorylation were mainly involved in neuron projection morphogenesis, cell polarity establishment and maintenance, and regulation of synapse structure or activity. Concurrently, proteins involved in the mRNA metabolic process, such as RNA splicing, exhibited increased phosphorylation (Figure 6B), suggesting broader molecular consequences of PP2Acα deficiency.

Global proteomic profiling identified 35,061 unique peptides, representing 6,196 proteins, of which 4,230 were quantifiable (Figure S9B). Differential expression analysis revealed 77 upregulated and 53 downregulated proteins in the Atoh1-cKO mice (|FoldChange (cKO/Ctrl)| > 1.2, *p*-value <0.05) (Figure 6C and Table S5). GO analysis indicated that upregulated proteins were predominantly enriched in neurodevelopmental processes (Figure 6D), such as neuron projection regeneration, glial cell differentiation, and oligodendrocyte differentiation.

Together, these findings demonstrate that PP2Acα in granule neurons plays a multifaceted role in cerebellar development by maintaining global phosphorylation homeostasis and regulating protein expression programs, thereby ensuring both structural integrity and intercellular function within the cerebellum.

## Discussion

**Figure 7:**
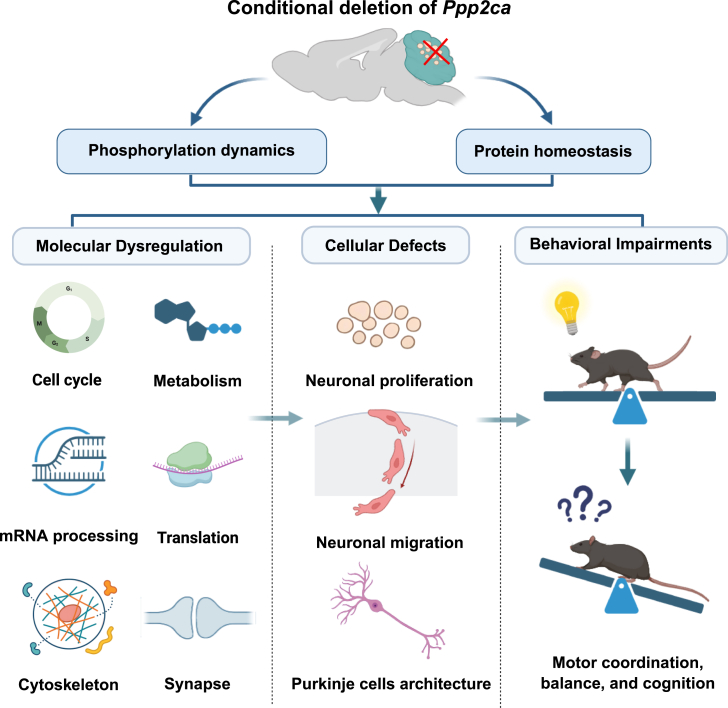
Schematic model of PP2Acα-regulated cerebellar development through multilayered control involving phosphorylation and protein homeostasis Conditional deletion of *Ppp2ca* in granule neurons resulted in the extensive dysregulation of phosphorylation-dependent pathways, spanning cell cycle regulation, metabolism, mRNA processing, translation, cytoskeletal organization, and synaptic signaling. This led to the reduced proliferation of granule neurons, delayed neuronal migration, and disrupted Purkinje cell architecture, which collectively resulted in deficits in motor coordination, balance, and cognition. Created in BioRender. Ma, C. (2026) https://BioRender.com/7y473le.

Previous clinical observations have linked mutations in *PPP2CA* to intellectual disability, developmental delay, ataxia, and structural brain abnormalities,^26^ suggesting a potential role for PP2Acα in cerebellar pathophysiology. However, prior mechanistic investigations of PP2Acα have been largely restricted to the cerebral cortex and hippocampus,^13^^,^^14^^,^^15^^,^^16^^,^^17^ leaving its functions in the cerebellum poorly defined. Our study directly addresses this gap by providing *in vivo* mechanistic evidence that granule neuron-specific loss of PP2Acα impaired cerebellar development, thereby establishing a mechanistic link between PP2Acα dysfunction and cerebellar disease phenotype. Specifically, the conditional deletion of *Ppp2ca* in granule neurons resulted in a cascade of cellular and structural defects, including reduced proliferation, M-phase accumulation, delayed neuronal migration, and disorganized Purkinje cell architecture, which collectively resulted in deficits in motor coordination, balance, and cognition. Mechanistically, phosphoproteomic and proteomic analyses revealed extensive dysregulation of phosphorylation-dependent pathways, spanning cell cycle regulation, mRNA processing, metabolism, translation, cytoskeletal organization, and synapse signaling (Figure 7). Importantly, these changes reflect both cell-autonomous effects within granule neurons and non-cell-autonomous effects propagating through the cerebellar circuitry, illustrating how granule neuron dysfunction can perturb the surrounding microenvironment and connectivity. Together, these results demonstrate that PP2Acα orchestrates diverse molecular programs whose disruption extends from the cellular to the circuit and behavioral level.

In primary granule neurons, we identified 29 hyperphosphorylated proteins as potential direct PP2Acα substrates, including both established PP2A targets (such as LMNA,^39^ MAP4,^40^ and MAP1B^41^) and previously uncharacterized candidates. Dysregulation of nuclear factors such as LMNA,^42^ HMGA1,^43^ DIDO1,^44^ and NCOA2^45^ likely perturbs chromatin structure and transcription, contributing to slowed proliferation or cell-cycle arrest. Notably, the altered phosphorylation of the transcription factor ZEB1, previously implicated in repressing polarity genes in granule neuron progenitors,^46^ may disrupt its transcriptional control over neuronal polarity and differentiation programs. Furthermore, the abnormal phosphorylation of cytoskeletal proteins (including MAP1A, MAP1B, MAP4, NAV1, MYO9B, FGD6, GIT1, and PLEC) provides a mechanistic basis for the observed migration defects by compromising microtubule and actin dynamics.^47^^,^^48^^,^^49^^,^^50^^,^^51^ Concomitantly, changes in vesicle trafficking proteins such as EPS15, STX7, UBAP2, BIN1, and PALM point toward impaired synaptic signaling, consistent with the deficits in motor coordination and balance observed in Atoh1-cKO mice.^52^ Together, these phosphorylation events provide mechanistic explanations for the observed cellular phenotypes in Atoh1-cKO mice.

Beyond these direct interactions, broader proteomic changes likely reflect indirect regulatory consequences or compensatory responses. Several of these alterations align with previously reported PP2A functions in both neuronal and non-neuronal contexts. First, the reduced phosphorylation of cell cycle regulators, such as CDK1^33^^,^^53^ and BRCA1,^54^ is consistent with the observed M-phase arrest phenotype, reinforcing PP2A’s conserved role in regulating cell cycle progression. Second, the upregulation of glycolytic enzymes (PGK1, ALDOA, LDHA, G6PI, PDK1, HXK2, PGAM1) suggests a metabolic shift toward glycolysis.^55^ While PP2A is a known regulator of glycolysis in pathological contexts such as cancer and diabetes,^56^^,^^57^ evidence in the brain has been limited. Notably, a recent report demonstrated that PP2A restrains glycolysis in microglia.^58^ Our findings extend this principle to cerebellar granule neurons, providing *in vivo* evidence that PP2Acα similarly regulates glycolytic reprogramming in developing neurons. Third, coordinated downregulation of ribosomal proteins and biogenesis factors suggests that PP2Acα is required for sustaining translational capacity in granule neurons.^59^ Although PP2A is a known regulator of protein synthesis through pathways such as mTORC1-S6K and 4E-BP1,^60^^,^^61^ its role in neuronal translation has remained largely unexplored, and our data provide *in vivo* evidence supporting this function in the developing cerebellum. Finally, hyperphosphorylation of proteins involved in cytoskeleton organization and cell projection, including both well-known PP2A substrates (Tau,^62^ CRMP2;^15^ Vimentin^63^) and candidates identified in this study-provides further evidence that PP2Acα is indispensable for neuronal migration and layering. Collectively, these phosphorylation changes converge on signaling pathways that regulate granule neuron proliferation, migration, and differentiation, thereby providing a mechanistic framework linking PP2Acα loss to the structural and functional defects observed in the cerebellum.

At the circuit level, Purkinje cells displayed disorganized dendrites despite PP2Acα deletion being restricted to granule neurons, highlighting non-cell-autonomous effects. In line with these findings, the pharmacological inhibition of PP2A in Purkinje cells has been shown to induce LTD by enhancing AMPA receptor phosphorylation and declustering at granule cell-Purkinje cell synapses,^20^ underscoring the critical role of PP2A in maintaining granule neuron-Purkinje cells connectivity. Moreover, cerebellar LTP at parallel fiber-Purkinje cell synapses critically depends on coordinated activities of PP1, PP2A, and PP2B.^11^ Consistent with this, the disruption of translational control by 4E-BP deletion has been reported to convert parallel fiber-Purkinje cell LTD into LTP as a result of enhanced PP2A activity, which can be reversed by PP2A inhibition,^21^ further supporting that maintaining proper PP2A activity is crucial for balanced cerebellar plasticity. Together with our findings, these studies suggest that PP2Acα acts bidirectionally within the cerebellar circuit, where perturbations in either granule cells or Purkinje cells converge on synaptic dysfunction and altered plasticity. To further explore the molecular basis underlying these circuit-level changes, we examined how PP2Acα deletion reshaped the cerebellar phospho-signaling landscape. Proteins with decreased phosphorylation were involved in neuron projection morphogenesis, polarity, and synapse organization. Conversely, upregulated proteins were enriched in pathways associated with adult behavior, synaptic signaling, and associative learning, aligning with the observed cognitive impairments and supporting the cerebellum’s role in higher-order functions. Intriguingly, although PP2Acα deletion is expected to increase the phosphorylation of its direct substrates, many proteins showed decreased phosphorylation. This apparent paradox likely reflects compensatory feedback mechanisms within the cerebellar network. Specifically, the loss of PP2Acα in granule cells may non-cell-autonomously alter downstream kinase/phosphatase activities in neighboring cells (e.g., Purkinje cells or glia). These complex network effects, together with the cellular heterogeneity of cerebellar tissue, collectively shape the global phosphorylation landscape following PP2Acα loss.

In conclusion, our study identifies PP2Acα as a key regulator of cerebellar development, coordinating multiple molecular programs in granule neurons and indirectly influencing Purkinje neurons and cerebellar circuitry. Loss of PP2Acα triggers a cascade of defects that culminate in motor and cognitive impairments resembling human *PPP2CA*-related disorders. These findings not only advance our understanding of cerebellar biology but also open new avenues for exploring PP2Acα-dependent mechanisms in neurodevelopmental disorders.

## Resource availability

### Lead contact

Further information and requests for resources and reagents should be directed to and will be fulfilled by the lead contact, Yamei Niu: niuym@ibms.pumc.edu.cn (Y.N.).

### Materials availability

Mouse models generated in this study are available from the lead contact upon request.

### Data and code availability

• Original data have been deposited on Mendeley Data (10.17632/htf9g6dw78.1) and are publicly available as of the date of publication.
• Data: The proteomics and phosphoproteomic data from this study have been deposited in the iProX, (no. IPX0014442000, https://www.iprox.cn//page/project.html?id=IPX0014442000) and are publicly available as of the date of publication.
• This article does not report original code.
• Any additional information required to reanalyze the data reported in this article is available from the lead contact upon request.

### Limitations of the study

Despite these advances, several important questions remain. First, the direct molecular interactions between PP2Acα and its candidate substrates require experimental validation, and the precise mechanistic links between specific phosphorylation changes and the observed cellular and structural defects remain to be fully elucidated, which represent an important direction for future investigation. Second, the balance between granule neuron-intrinsic and extrinsic contributions to other cell phenotypes warrants further dissection, potentially through cell-type-specific rescue approaches. Third, the roles of PP2Acα in other cerebellar cell types, including Purkinje cells and glia, remain to be explored. Addressing these issues collectively will deepen our understanding of PP2Acα and its contributions to cerebellar development and pathology.

## Acknowledgments

We thank Professors Xiang Gao and Jiangang Gao for providing *Ppp2ca* floxed mice and *Atoh1-Cre* mice. This study was supported in part by Noncommunicable Chronic Diseases-10.13039/501100018537National Science and Technology Major Project(Grant No. 2023ZD0507100) and the 10.13039/501100005150Chinese Academy of Medical Sciences (CAMS) Innovation Fund for Medical Sciences (2021-I2M-1-020).

## Author contributions

N.Y., T.W.M., and L.C.: conceptualization, investigation, resources, writing-original draft, writing-review and editing, project administration, and funding acquisition. L.Y. and D.J.: methodology, software, validation, formal analysis, data curation, writing-original draft, and writing-review and editing. L.S., W.Q., L.Q., and L.A.: methodology, validation, and writing-review and editing. F.Y.: data curation, software, and writing-review and editing. The work reported in the article has been performed by the authors, unless clearly specified in the text.

## Declaration of interests

The authors declare no competing interests.

## Declaration of generative AI and AI-assisted technologies in the writing process

During the preparation of this work, the authors used ChatGPT (OpenAI) to assist with language refinement, clarity, and expression. All content was reviewed and edited by the authors, who take full responsibility for the final publication.

## STAR★Methods

### Key resources table

**Table:** 

REAGENT or RESOURCE	SOURCE	IDENTIFIER
**Antibodies**		

Mouse anti-Nestin	Millipore	Cat#MAB353; RRID: AB_94911
Mouse anti-NeuN	Millipore	Cat#MAB377; RRID: AB_2298772
Mouse anti-NeuN	Abcam	Cat#ab104224; RRID: AB_10711040
Mouse anti-Tubulin beta-3 (TUBB3)	Biolegend	Cat#801202; RRID: AB_2313773
Mouse anti-GFAP	CST	Cat#3670; RRID: AB_561049.
Rabbit anti-PP2Acα	ABclonal	Cat#A6702; RRID: AB_2767286
Rabbit anti-PP2A	Bethyl	Cat#A300-732A; RRID: AB_2237618
Mouse anti-D28K	Sigma	Cat#C9848; RRID: AB_476894
Rabbit anti-Ki67	Abcam	Cat#ab66155; RRID: AB_1140752
Mouse anti-Phospho-Histone H3 (Ser10)	CST	Cat#9706S; RRID: AB_331748.
Mouse anti-5-bromo-2′-deoxyuridine (BrdU)	Proteintech	Cat#66241-1-Ig; RRID: AB_2881630
Mouse anti-α-Tubulin	Sigma	Cat#T6074; RRID: AB_477582
Goat anti-rabbit Alexa Fluor 488	Invitrogen	Cat#A-11008; RRID: AB_143165
Goat anti-mouse Alexa Fluor 488	Invitrogen	Cat#A-11001; RRID: AB_2534069
Goat anti-mouse Alexa Fluor 594	Invitrogen	Cat#A-11005; RRID: AB_2534073

**Chemicals, peptides, and recombinant proteins**		

DAPI	Sigma	Cat#F6057
Protease inhibitor cocktail	Roche	Cat#04693159001
Phosphatase inhibitor cocktail	Roche	Cat#04906837001
TriReagent	Sigma	Cat#T9424
Neurobasal A Culture Medium	Gibco	Cat#10888022
NeuroCult^TM^ Basal completed medium (Mouse & Rat)	Stem Cell Technologies	Cat#5702
5-Bromo-2′-deoxyuridinesingle-pulse	Beyotime	Cat#ST1056
ReverTra Ace qPCR RT Master Mix	TOYOBO	Cat#FSQ-301
2× SYBR Green PCR master mix	TOYOBO	Cat#QPS-201
BCA protein assay kit	GenStar	Cat#E162-05
Polyvinylidene fluoride membranes	Millipore	Cat#IEVH00005
Chemistar™ High-sig ECL Western Blotting Substrate	Tanon	Cat#180-5001

**Oligonucleotides**		

*Ppp2ca-*forward: 5′-tagcccatgcctttaatctcagagc-3′	Lin, Lin et al.^14^	N/A
*Ppp2ca-*reverse: 5′-cactcgtcgtagaacccataaacc-3′	Lin, Lin et al.^14^	N/A
*Cre*-forward: 5′-cggtcgatgcaacgagtgatgagg-3′	Lin, Lin et al.^14^	N/A
*Cre*-reverse: 5′-ccagagacggaaatccatcgctcg-3′	Lin, Lin et al.^14^	N/A
*β2m*-forward: 5′-caccggagaatgggaagccgaa-3′	Lin, Lin et al.^14^	N/A
*β2m-*reverse: 5′-tccacacagatggagcgtccag-3′	Lin, Lin et al.^14^	N/A
*Ppp2ca-*forward-real-time PCR: 5′-ctcgtcgtaccccagactac-3′	Lin, Lin et al.^14^	N/A
*Ppp2ca-*reverse-real-time PCR: 5′-gcacatcttttggtccgtgt-3′	Lin, Lin et al.^14^	N/A
*U1*-forward-real-time PCR: 5′-atacttacctggcaggggagatacc-3′	This paper	N/A
*U1-*reverse-real-time PCR: 5′- agcctcgccctgggaaaa-3′	This paper	N/A

**Experimental models: Organisms/strains**		

*Ppp2ca* floxed mice (C57BL/6)	Lin, Lin et al.^14^^,^^64^	N/A
*Nestin-Cre* mice (C57BL/6)	Lin, Lin et al.^14^	N/A
*Atoh1-Cre* mice (C57BL/6)	Men et al.^28^	N/A

**Deposited data**		

Single-cell RNA-seq data: A transcriptomic atlas of the mouse cerebellum	Kozareva V et al.^27^	GSE165371, doi: https://doi.org/10.1038/s41586-021-03220-z
Proteomics and phosphoproteomics	This paper	iProX: IPX0014442000, https://www.iprox.cn//page/project.html?id=IPX0014442000
Data obtained in this study	This paper	Niu, Yamei (2025), “PP2Aca regulates cerebellar development”, Mendeley Data, V1, doi: https://doi.org/10.17632/htf9g6dw78.1

**Software and algorithms**		

Panoramic MIDI II digital slide scanner	3DHistech	Pannoramic MIDI II Digital Scanner · 3DHISTECH
Ethovision XT 8	Noldus Information Technology	EthoVision XT | Video tracking software | Noldus, RRID:SCR_000441
GraphPad Prism 9.0	GraphPad Software	https://www.graphpad.com/, RRID:SCR_002798
ImageJ	NIH	https://imagej.net/ImageJ, RRID: SRC_003070
BioRender	https://biorender.com/	Scientific Image and Illustration Software | BioRender, RRID:SCR_018361

### Experimental model and study participant details

#### Animals

The *Ppp2ca* floxed mice (C57BL/6) were provided by Prof. Xiang Gao from Model Animal Research Center and have been previously described.^14^^,^^64^ *Nestin-Cre* mice (C57BL/6) were obtained from the Jackson Laboratory.^14^ *Atoh1-Cre* mice (C57BL/6) were provided by Prof. Jiangang Gao from the School of Life Sciences, Shandong University.^28^ These mice were interbred to obtain *Ppp2ca*^fl/fl^ *Nestin-Cre*^+^ mice and *Ppp2ca*^fl/fl^ *Atoh1-Cre*^+^ mice (denoted as Nestin-cKO and Atoh1-cKO), *Ppp2ca*^fl/+^ *Atoh1-Cre*^+^ mice (denoted as Atoh1-cHet) and control group (*Ppp2ca*^fl/fl^ *Nestin-Cre*^-^; *Ppp2ca*^fl/+^ *Nestin-Cre*^-^; *Ppp2ca*^+/+^ *Nestin-Cre*^-^; *Ppp2ca*^+/+^ *Nestin-Cre*^+^; *Ppp2ca*^fl/fl^ *Atoh1-Cre*^-^; *Ppp2ca*^fl/+^ *Atoh1-Cre*^-^; *Ppp2ca*^+/+^ *Atoh1-Cre*^-^; *Ppp2ca*^+/+^ *Atoh1-Cre*^+^). Data from both sexes were pooled for all analyses. Genotyping was performed by using genomic DNA extracted from mouse tails as described previously.^64^ Primer sequences are shown in key resources table.

All the above animals are raised in the individual ventilation cages within the SPF-level barrier facilities of IBMS/PUMC experimental animal center. Environmental conditions were maintained at a constant temperature and humidity, with a 12-h light/dark cycle. Animals had *ad libitum* access to food and water. All animal experiments and euthanasia were performed in compliance with the guidelines of IBMS/PUMC Animal Care and Use Committee and approved under the institutional protocol (approval number: ACUC-A01-2019-026).

#### Primary culture of mouse cerebellar granulosa cells

Cerebellar tissues from P5–P6 mice were digested with 0.25% Trypsin (Gibco, 15400-054) in Hanks' Balanced Salt Solution (HBSS) (Gibco, 14025076). The digestion was terminated with Neurobasal A Culture Medium (Gibco, 10888022) and resuspended in NeuroCult Basal completed medium (Mouse & Rat) (Stem Cell Technologies, 5702) for culture at 37°C with 5% CO_2_.

### Method details

#### Histology and immunostaining analysis

Mice were anesthetized by intraperitoneal injection of tribromoethanol solution and then perfused with 4% paraformaldehyde (PFA). Whole brains were dissected and post-fixed in 4% PFA for 48–72 h at 4°C, followed by dehydration and paraffin embedding. Serial 4-μm-thick paraffin sections were prepared for H&E staining or immunohistochemistry analysis. Primary antibodies used are listed in key resources table. Images of H&E and immunohistochemical staining were acquired using a Panoramic MIDI II digital slide scanner (3D Histech).

For cryosections, tissues were fixed in 4% PFA, dehydrated in 30% sucrose, and embedded with O.C.T. compound (Tissue-Tek, Sakura, USA, 4583). Cryosections (15–60 μm thick) were prepared using a cryostat (Leica Microsystem) for immunofluorescence staining. Slides were mounted with Fluoroshield with DAPI (Sigma, F6057), dried at 4°C in the dark, and imaged using a confocal microscope (Zeiss LSM 780 and Nikon A1 HD25).

For 5-Bromo-2′-deoxyuridinesingle-pulse (BrdU, Beyotime, ST1056) labeling, mice received intraperitoneal injections (50 μg/g body weight) on P4 or P8. Brains were collected 2 h or 96 h post-injection for BrdU immunofluorescence as previously described.^65^ BrdU-labeled cells were quantified using ImageJ software (NIH).

Primary cerebellar granule cells were plated on poly-L-lysine-coated coverslip and then cultured for 4–6 days before fixation with 4% PFA (10–20 min, RT). Cells were permeabilized at room temperature with PBS solution containing 0.1% Triton X-100 and 0.5% NP-40 for 10 min prior to immunostaining.

RNA Isolation and Real-Time Quantitative PCR (RT-qPCR) Total RNA was extracted from cerebellar tissues and primary cells using TRIzol reagent (Sigma, T9424) according to the manufacturer’s protocol. ReverTra Ace qPCR RT Master Mix (TOYOBO, FSQ-301) was used to synthesize the first-strand cDNAs with random hexamers. qPCR was performed in 10 μL reaction containing StepOnePlusTM Real-time PCR instrument (Thermo Fisher Scientific), 1:10 diluted cDNA, 2× SYBR Green PCR master mix (TOYOBO, QPS-201), and 0.6 μM primers. Primer sequences are listed in key resources table.

#### Western blot analysis

Proteins were extracted using RIPA lysis buffer (20 mM HEPES pH 7.6, 20% glycerol, 0.5 M NaCl, 1.5 mM MgCl_2_, 0.2 mM EDTA pH 8.0, 0.5% NP-40, 1 mM DTT, 1 mM PMSF, 5 μg/mL leupeptin, 2 μg/mL aprotinin, 1 mM β-glycerophosphate, 1 mM Na_3_VO4, and 10 mM NaF) supplemented with protease (Roche, 04693159001) and phosphatase inhibitor cocktail (Roche, 04906837001). Protein concentrations were determined using by BCA protein assay (GenStar, E162-05). Equal amounts of protein were separated by SDS‒PAGE and transferred onto polyvinylidene fluoride membranes (Millipore, IEVH00005). After blocking with 5% non-fat milk in Tris-buffered saline with Tween 20 (TBS-T) for 1 h at room temperature, membranes were incubated with primary antibodies overnight at 4 °C, followed by incubation with HRP-conjugated secondary antibodies for 1 h at room temperature. Proteins were detected using Chemista High-sig ECL Western Blotting Substrate (Tanon, 180–5001) and imaged with a gel documentation system (Tanon, 5800). Antibodies used in this study are listed in key resources table.

#### Behavior tests

##### Hindlimb clasping

For the Hindlimb clasping test,^66^ mice were lifted by their tails and the position of their hind limbs was observed and scored for 10 s in a blinded manner. Unaffected mice consistently splayed their hindlimbs outward away from the abdomen, and this behavior would be scored 0. A score of 1 was given to mice that pulled one hindlimb hind legs partially toward their abdomen for more than 5 s. When this was observed for both hind limbs, the mouse received a score of 2. A score of 3 was assigned to mice that retracted both hind limbs completely for more than 50% of the observation time.

##### Balance beam experiment

The balance beam, 1 m long, 35 cm wide and 1.5 cm thick, was positioned 100 cm above the ground, and supported by two pillars, one on each side. Under quiet circumstances, mice are initially placed on the balance beam for walking training. The training is carried out 3–4 times a day, with an interval of at least 30 min between each session. The duration of the training spans 3–4 days. The entire procedure is video-recorded to monitor the state and time of the mice’s locomotion on the balance beam. The number of times the hind paw on one side drops during a single walk is counted, serving as an evaluation criterion.

##### Rotarod test

This experiment was executed by employing a small animal rod turning fatigue detector (Ugo Basile, 47650). A three-day training course was implemented, with each day comprising six training sessions separated by a minimum interval of 30 min. During the training, the rotational speed was adjusted in a specific manner which was gradually increased from 5 rpm to 30 rpm over a 120-s period. The training was considered complete when the mice could stay on the rod without falling for 120 s. For the testing phase, the rotational speed progressively increased from 5 rpm to 40 rpm over a duration of 5 min. The time elapsed until the mice fell off the rod and the rotational speed of the rod at the exact moment of their fall were recorded. The results of three individual tests were averaged for analysis.

##### Y maze test

For the Y maze test, spontaneous alternations were measured using a Y-shaped maze (30 cm length × 5 cm width × 12 cm height per arm, with a 120° angle between each arm). Mice were randomly placed in one of the arms and allowed to freely explore for 8 min, with the camera system recording their behavioral changes. After each trial, the maze was cleaned with water and a dry cloth to prepare for the next animal. An alternation was counted each time an animal entered all three arms consecutively in overlapping triplet sets. The total arm entries and the numbers of alternation were recorded to calculate the percentage of spontaneous alternations: % Alternation = [Number of Alternations/(Total number of arm entries - 2)] ∗ 100%.

##### Morris water maze test

The Morris water maze was performed as previously described.^67^ Briefly, a transparent 12 cm-diameter platform was placed 1 cm below the water surface at a fixed position. During the training phase, each mouse underwent continuous training for 3 days with 4 trials/day, lasting 90 s each or until the mouse found the hidden platform. On the probe trial day, the platform was removed, and mice were allowed to swim for 60 s, starting from the quadrant opposite to the location of the platform. After the mice became adapted to swimming, the platform was raised above the water surface, and the mice were given 60 s to search for it. The time taken by the mice to find the platform was recorded to eliminate the influence of differences in vision and motor ability on the experiment. Behavioral parameters were recorded using a video camera positioned above the water tank, and data were analyzed using Ethovision XT 8 software (Noldus).

#### IBT-based quantitative proteomic and phosphoproteomic analysis in primary granule neurons

Pooled proteins from two independent sets of primary cerebellar granule cells from P8 mice were prepared using SDS-free protein lysis buffer (with 1× protease inhibitor, phosphatase inhibitor and 10 mM DTT) and quantified by Bradford assay. After reduction in 10 mM DTT, thiol blocking in 55 mM IAM, proteins were digested with trypsin (0.01 μg/μL in 25 mM NH_4_HCO_3_). A 5-fold volume of 50% acetonitrile (ACN) was added the next day, followed by vortexing and centrifugation. The peptide fragment after digestion and desalting was dissolved in 0.2 M TEAB solution to obtain a peptide concentration of 4 μg/μL, and labeled with Isobaric Tags (IBT) reagent. Component separation was performed using a High PH RP cartridge (Thermo Scientific Pierce). The dried peptides were reconstituted with 300 μL of 0.1% TFA and were reconstituted with mobile phase A (2% ACN, 0.1% FA). After centrifuged, the supernatant was taken for injection. Separation was performed by Thermo UltiMate 3000 UHPLC. The sample was first enriched in trap column and desalted, and then entered a self-packed C18 column (75 μm internal diameter, 3 μm column size, 25 cm column length) and separated at a flow rate of 300 nL/min by the following effective gradient: 0–5 min, 5% mobile phase B (98% ACN, 0.1% FA); 5–45 min, mobile phase B linearly increased from 5% to 25%; 45–50 min, mobile phase B increased from 25% to 35%; 50–52 min, mobile phase B rose from 35% to 80%; 52–54 min, 80% mobile phase B; 54–60 min, 5% mobile phase B. The nanoliter liquid phase separation end was directly connected to the mass spectrometer.

The peptides separated by liquid phase chromatography were ionized by a nanoESI source and then passed to a tandem mass spectrometer Q-Exactive HF X (Thermo Fisher Scientific, San Jose, CA) for DDA (Data Dependent Acquisition) mode detection.

Proteome Discoverer 1.4 was used to integrate MASCOT 2.3 for phosphoproteome identification. Percolator was used to reprocess the results of Mascot search, and the trusted peptide was obtained by using FDR ≤0.01 of the spectra as the filtering condition. Proteome Discoverer integrated phosphoRS were used to score the phosphorylation sites of the identified phosphopeptides, and the trusted phosphorylation sites were obtained by using phosphoRS probability ≥0.75 as the filtering condition. Finally, function annotation and Motif analysis were performed for the phosphoproteins corresponding to the trusted phosphorylated sites. Two filtration criteria (FoldChange >1.2 or <0.833, and *p*-value <0.05) were used to get differential phosphopeptides. We performed repeatability analysis (CV distribution) and cluster analysis on significantly differential phosphopeptides, and enrichment analysis and Motif analysis of phosphorylated sites on the differential phosphoproteins corresponding to significantly differential phosphopeptides.

#### TMT-labeling quantitative proteomic and phosphoproteomic analysis in mouse cerebellar tissue samples

Pooled proteins from three individual cerebellar tissues of P8 Ctrl and Atoh1-cKO mice were prepared using lysis buffer (8 M urea, 1% Protease inhibitor cocktail) and quantified with BCA kit.

Then, the lysate was reduced (5 mM DTT) for 30 min at 56°C, alkylated (11 mM iodoacetamide) for 15 min at room temperature in darkness. The protein sample was then diluted by adding 100 mM TEAB to urea concentration less than 2 M. Finally, trypsin was added at 1:50 trypsin-to-protein mass ratio for the first digestion overnight and 1:100 trypsin-to-protein mass ratio for a second 4 h-digestion. The peptides were labeled with Tandem Mass Tag (TMT) reagents from TMT kit (Thermo Fisher Scientific) according to the manufacturer’s instructions. Then, tryptic peptides were fractionated by high pH reverse-phase High-Performance Liquid Chromatography (HPLC) using Thermo Betasil C18 column (5 μm particles, 10 mm ID, 250 mm length). For affinity enrichment of phosphopeptides, the peptides were incubated with Ti4+-IMAC microspheres suspension in loading buffer (50% acetonitrile/6% trifluoroacetic acid) with vibration. The IMAC microspheres were washed with 50% acetonitrile/6% trifluoroacetic acid and 30% acetonitrile/0.1% trifluoroacetic acid to remove nonspecifically adsorbed peptides. Eluted phosphopeptides or tryptic peptides were analyzed by LC-MS/MS using an EASY-nLC 1000 UPLC system followed by tandem mass spectrometry (MS/MS) in Q ExactiveTM Plus (Thermo Fisher Scientific) coupled online to the UPLC.

The MS/MS data were compared with the Swissprot_Mouse database using Maxquant search engine (version 1.5.2.8) using the following search criteria: (i) Trypsin/P was specified as cleavage enzyme allowing up to 4 missing cleavages. (ii) The mass tolerance for precursor ions was set as 20 ppm in First search and 5 ppm in Main search, and the mass tolerance for fragment ions was set as 0.02 Da. (iii) Alkylation on Cys was specified as fixed modification and Acetylation and deamination of N-terminus, oxidation on Met, phosphorylation of Ser, Thr and Tyr were specified as variable modifications. FDR was adjusted to <1% and minimum score for modified peptides was set >40. The site localization probability was set as > 0.75. The up-regulated and down-regulated thresholds were set at 1.20 and 0.83, respectively.

#### Gene ontology (GO) analysis

Gene Ontology (GO) terms were assigned using Metascape,^68^ UniProt-GOA database^69^ and InterProScan^70^ for unannotated protein’s. Two-tailed Fisher’s exact tests were employed for enrichment analysis of differentially phosphorylated (significant if *p*-value <0.05).

### Quantification and statistical analysis

#### Statistical analysis

All statistical analysis was assessed by unpaired two-tailed Student’s *t* test or one-way ANOVA using GraphPad Prism 9 software, as specified in the figure legends. Data are presented as mean ± standard error of the mean (SEM) and n represents the number of biological replicates, unless otherwise stated. *p*-value <0.05 was considered statistically significant.

## Supplemental information

Document S1. Figures S1–S9

Table S1. List of proteins with increased or decreased phosphorylation levels in primary granule neurons from Atoh1-cKO mice

Table S2. List of differentially expressed proteins in primary granule neurons from Atoh1-cKO mice

Table S3. List of 29 candidate substrate proteins of PP2Acα in the primary granule neurons

Table S4. List of proteins with increased or decreased phosphorylation levels in cerebellar tissues from Atoh1-cKO mice

Table S5. List of differentially expressed proteins in cerebellar tissues from Atoh1-cKO mice
