# Supplementary material for: PP2Acα regulates cerebellar development via phosphorylation-dependent neuronal programs
Source: iScience. 2025 Dec 5;29(1):114352. doi: 10.1016/j.isci.2025.114352 (PMC12774704; doi:10.1016/j.isci.2025.114352)
Supplement: Document S1. Figures S1–S9 [file mmc1.pdf]

iScience, Volume *29*

Supplemental information

PP2Acα regulates cerebellar development via phosphorylation-dependent neuronal programs

Yifan Li, Jing Ding, Simeng Liu, Qiao Wu, Yujie Fu, Qing Li, An Lv, Chunying Liu, Wei-Min Tong, and Yamei Niu

Supplemental information

Document S1. Figures S1-S9

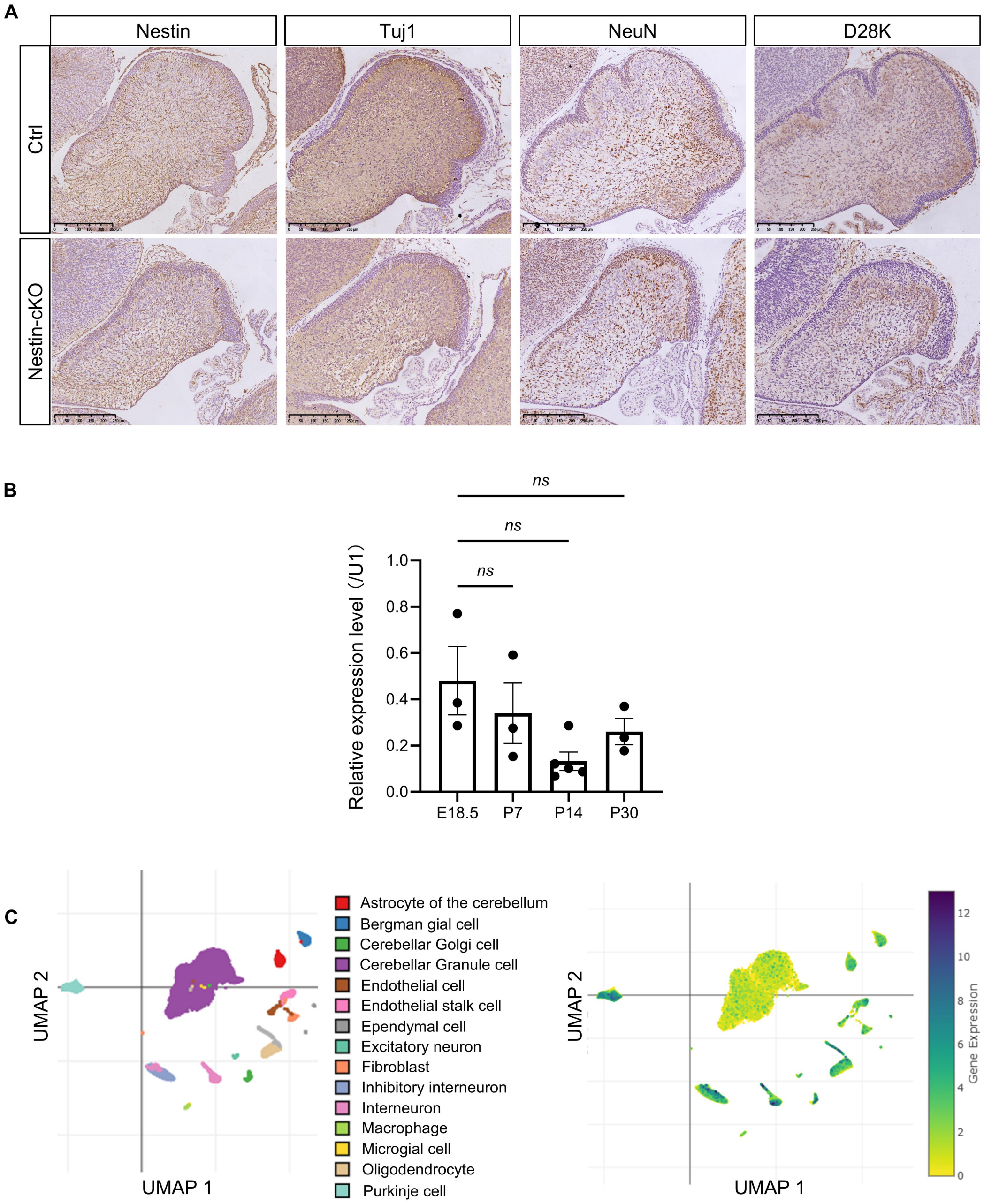

Figure S1. Cerebellar developmental abnormalities of Nestin-cKO mice at E18.5 and the age- and cell-type-specific expression patterns of *Ppp2ca* (related to Figure 1).

(A) Immunohistochemical staining of Nestin, Tuj1, NeuN and D28K in the cerebellum of Ctrl and Nestin-cKO mice. n = 4. Scale bar, 250 μm. (B) RT-qPCR analysis showing *Ppp2ca* expression in the cerebellum at different developmental stages (E18.5, P7, P14 and P30). n=3. *U1* served as internal control. Data are represented as mean ± SEM. *ns*: non-significant (two-sided one-way ANOVA). (C) UMAP plots showing clustering of cerebellar cell types in adult mice (Left) and the corresponding expression of *Ppp2ca* (Right). Data obtained from the Single Cell Portal.

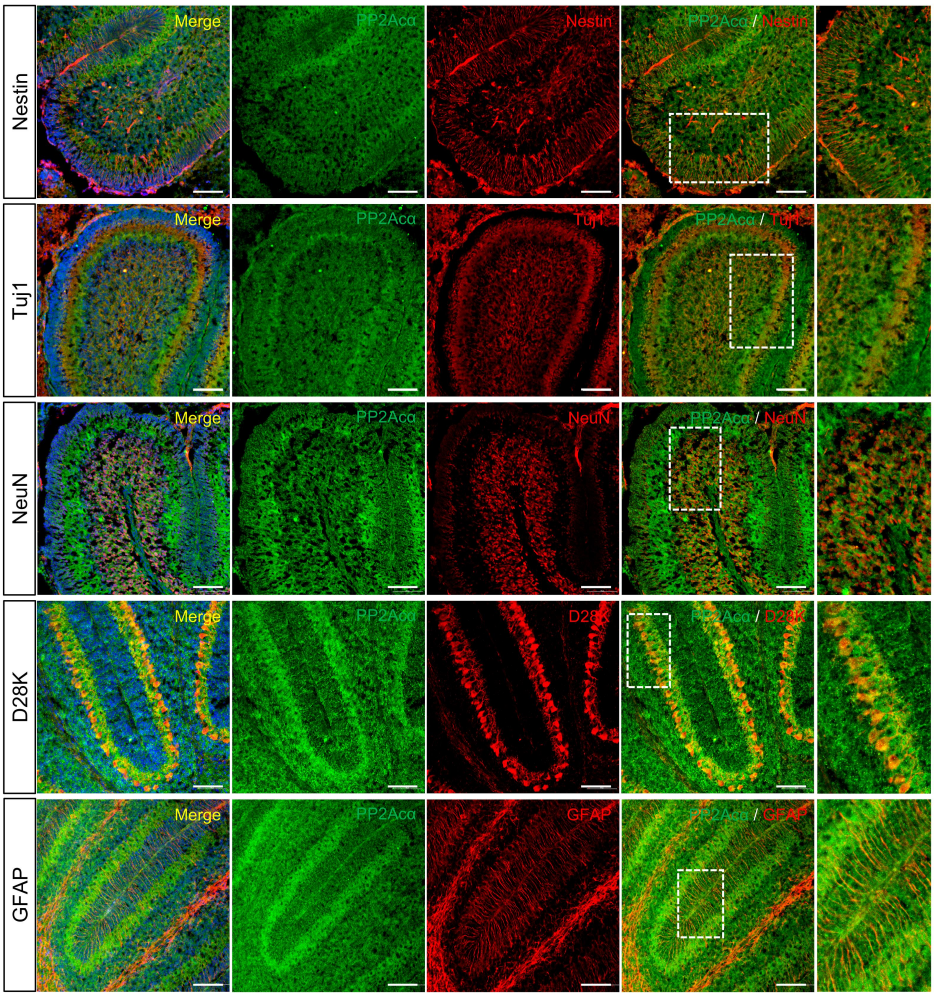

Figure S2. Cell-type-specific expression of PP2Acα in the cerebellum from P8 mouse (related to Figure 1).

Co-immunostaining of PP2Acα (Green) with stage-specific neuronal markers (Nestin, Tuj1, and NeuN, Red), the Purkinje cell marker D28K (Red) and the astrocyte marker GFAP (Red). Enlarged views of the boxed regions are shown in the right panels. n = 3. Scale bar, 100 μm.

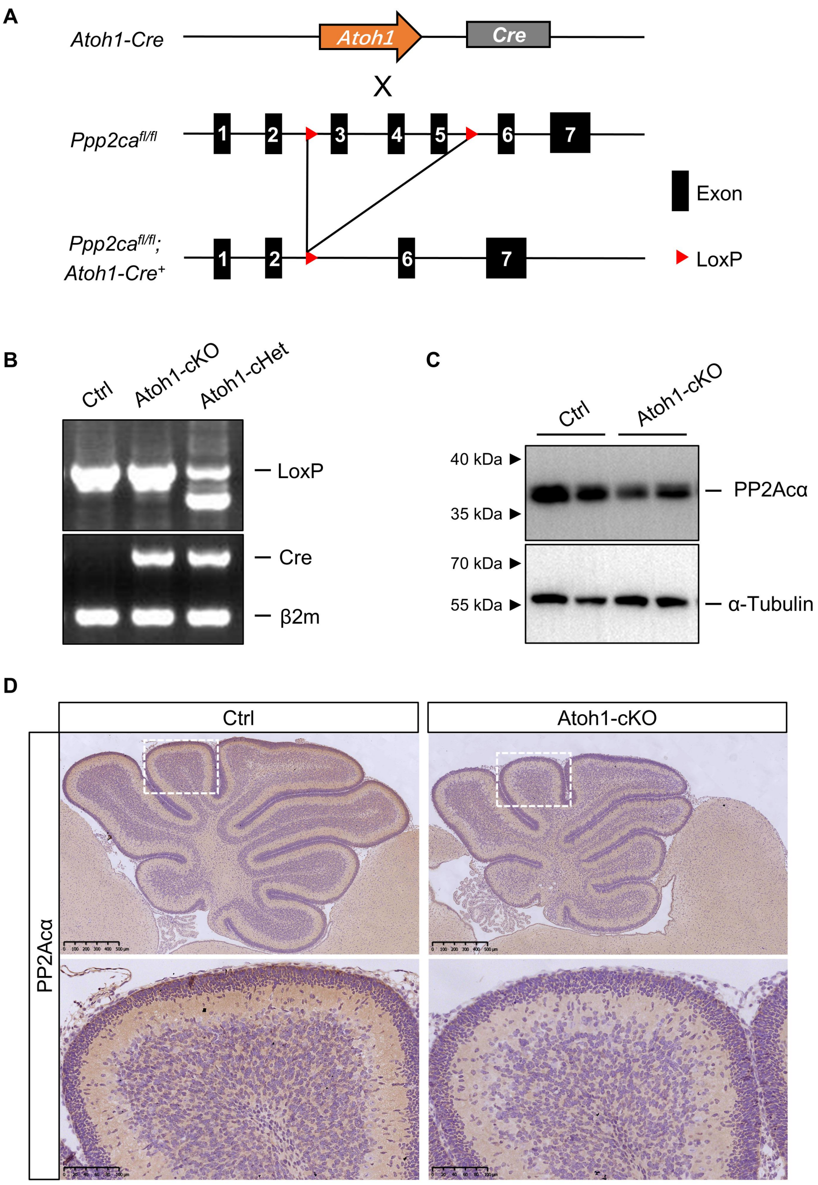

E

Genotype	Numbers	Distribution (%)
Ctrl	31	48
Atoh1-cHet	12	19
Atoh1-cKO	21	33
Total	64	100

Figure S3. Generation and validation of *Ppp2ca*$^{fl/fl}$; *Atoh1-Cre*$^{+}$ mice. (related to Figure 2)

(A) Schematic illustration of *Ppp2ca*$^{fl/fl}$ and *Atoh1-Cre*$^{+}$-mediated recombination. (B) Representative PCR genotyping results of *Ppp2ca*$^{fl/fl}$; *Atoh1-Cre*$^{+}$ mice. *β2m* served as internal control. (C) Western blot analysis showing reduced PP2Acα protein expression in the cerebellum of Atoh1-cKO mice. α-tubulin served as loading control. (D) Immunohistochemical staining showing condition ablation of PP2Acα in the cerebellum. n=3. Scale bars, 100 μm and 500 μm. (E) Genotype distribution of *Ppp2ca*$^{fl/fl}$ mouse hybrid offspring (up to 1.5 years of age).

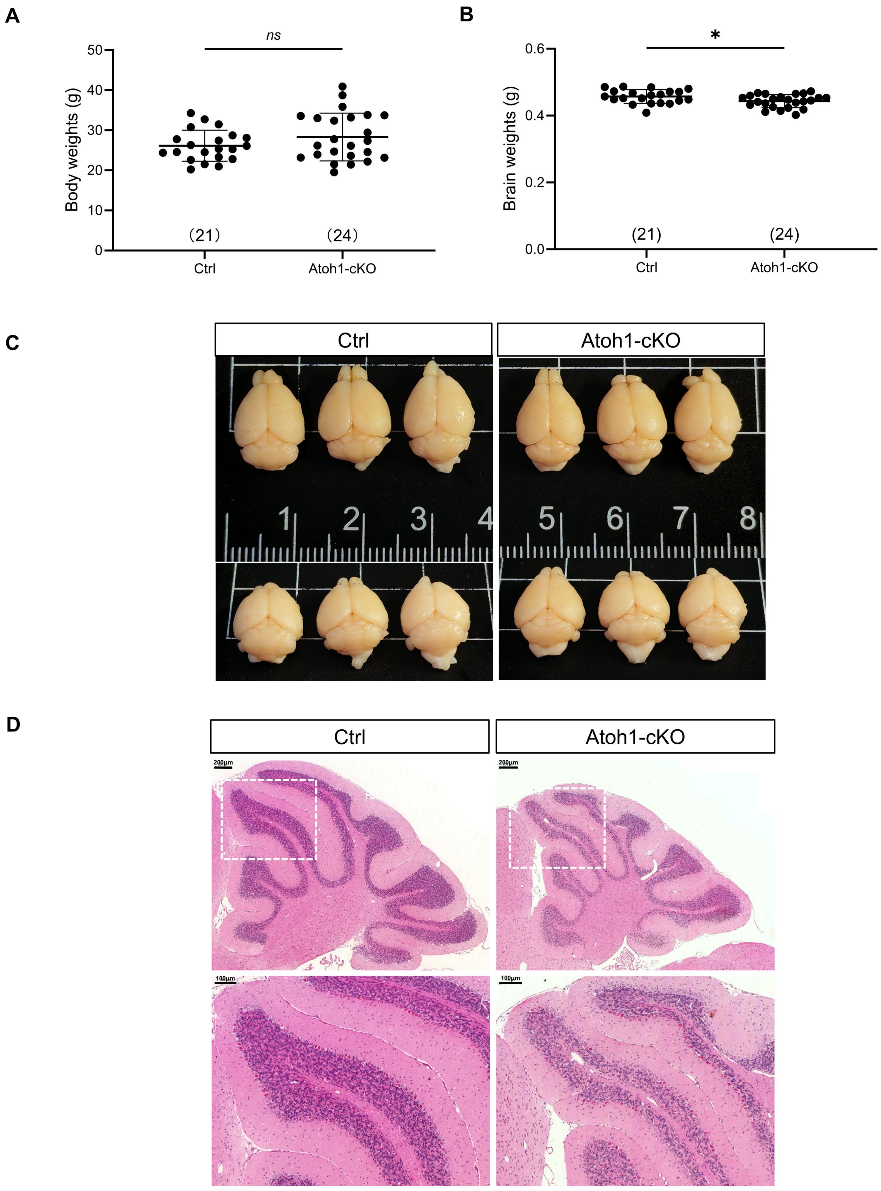
A
ns
Body weights (g)
50
40
30
20
10
0
(21)
(24)
Ctrl
Atoh1-cKO
B
*
Brain weights (g)
0.6
0.4
0.2
0.0
(21)
(24)
Ctrl
Atoh1-cKO
C
Ctrl
Atoh1-cKO
D
Ctrl
Atoh1-cKO

Figure S4. Cerebellar developmental defects in 3-month-old Atoh1-cKO mice (related to Figure 2).

(A) Quantitative comparison of body weights of individual Ctrl and Atoh1-cKO mice. (B) Quantitative comparison of brain weights of individual Ctrl and Atoh1-cKO mice. Data are represented as mean ± SEM. $*p < 0.05$ and *ns*.: non-significant (two-sided unpaired t-test). (C) Representative brain images of Ctrl and Atoh1-cKO mice. (D) H&E staining of cerebellar sections from Ctrl and Atoh1-cKO mice. n = 3. Scale bars, 100 μm and 200 μm.

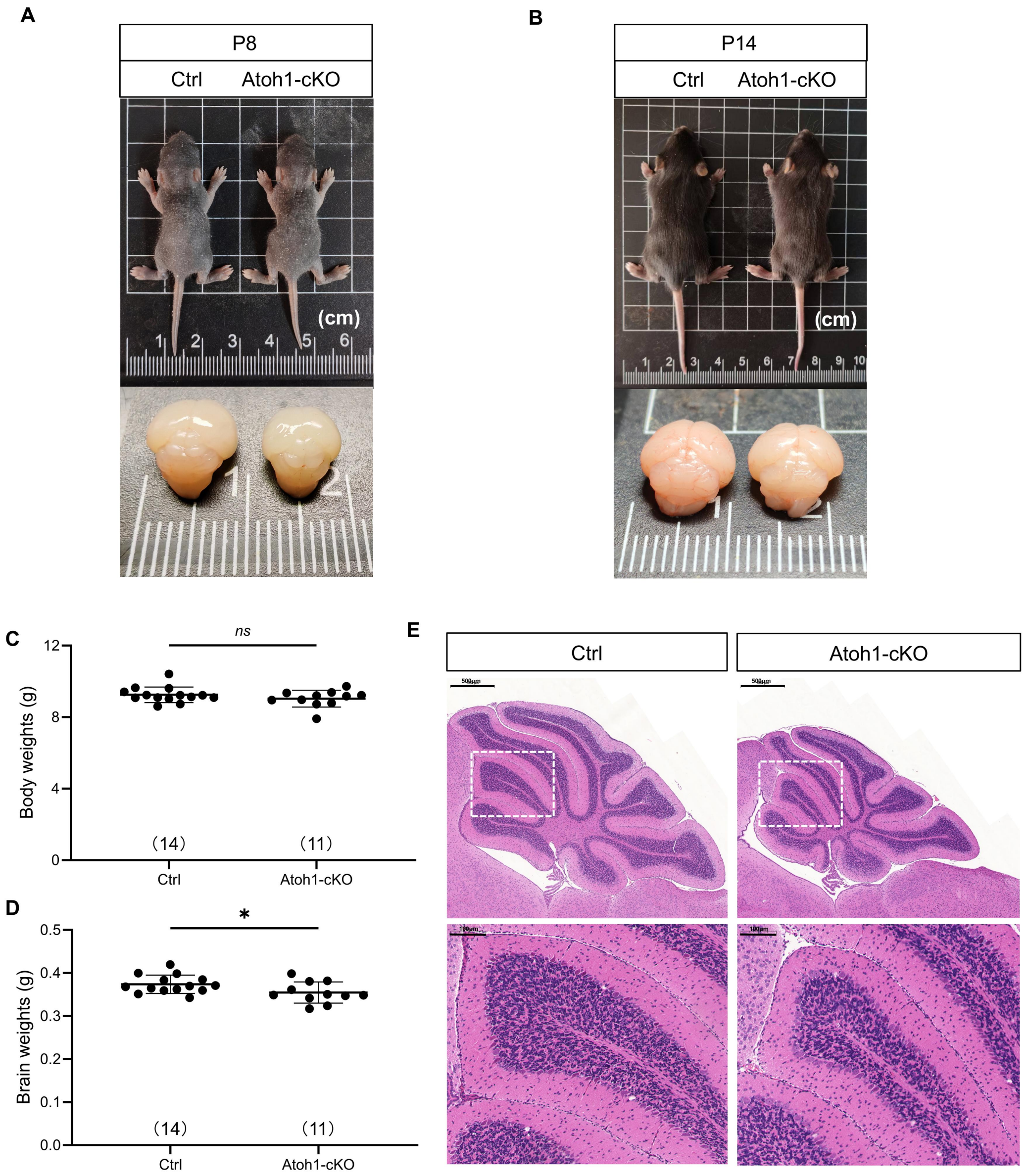

Figure S5. Morphology defects in P8 and P14 Atoh1-cKO mice (related to Figure 3).

(A-B) Representative whole-body and brain images of Ctrl and Atoh1-cKO mice at P8 (A) and P14 (B). (C-D) Quantitative comparison of body weights (C) and brain weights (D) of individual Ctrl and Atoh1-cKO mice at P14. Data are represented as mean ± SEM. *$p < 0.05$ and *ns*.: non-significant (two-sided unpaired t-test). (E) H&E staining of cerebellar sections from Ctrl and Atoh1-cKO mice at P14. n = 4. Scale bars, 100 μm and 200 μm.

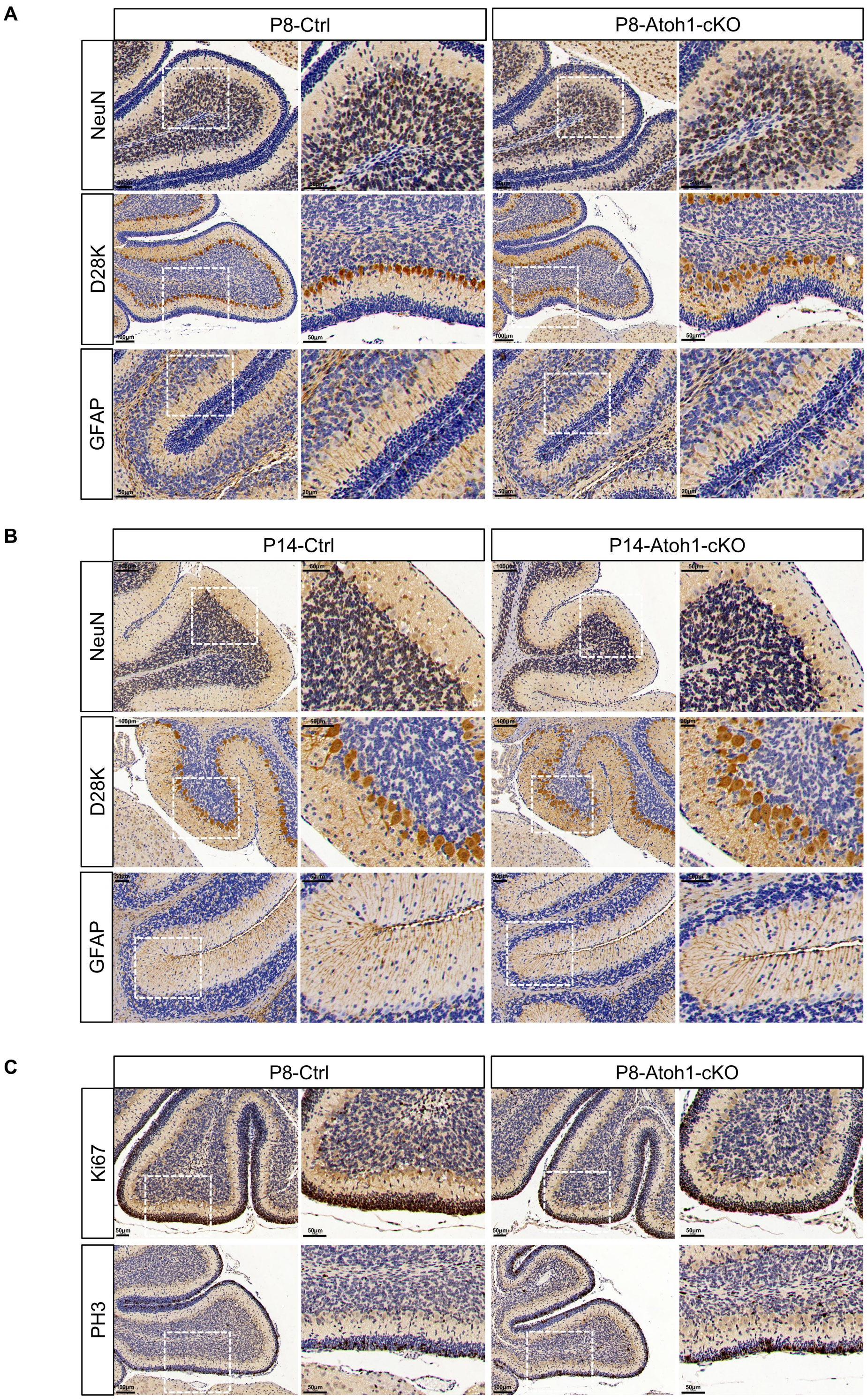
A
P8-Ctrl
P8-Atoh1-cKO
NeuN
D28K
GFAP
B
P14-Ctrl
P14-Atoh1-cKO
NeuN
D28K
GFAP
C
P8-Ctrl
P8-Atoh1-cKO
Ki67
PH3

Figure S6. Cell type-specific alterations in the cerebellum of P8 and P14 Atoh1-cKO mice (related to Figure 3).

(A-B) Immunohistochemical staining of NeuN, D28K and GFAP in the cerebellum of Ctrl and Atoh1-cKO mice at P8 (A) and P14 (B). P8, n = 5. P14, n = 3. Enlarged views of the boxed region are shown in the right panel. Scale bars, 20 μm, 50 μm and 100 μm. (C) Immunohistochemical staining of Ki67 and PH3 in the cerebellum of Ctrl and Atoh1-cKO mice at P8. n = 5. Scale bars, 50 μm and 100 μm.

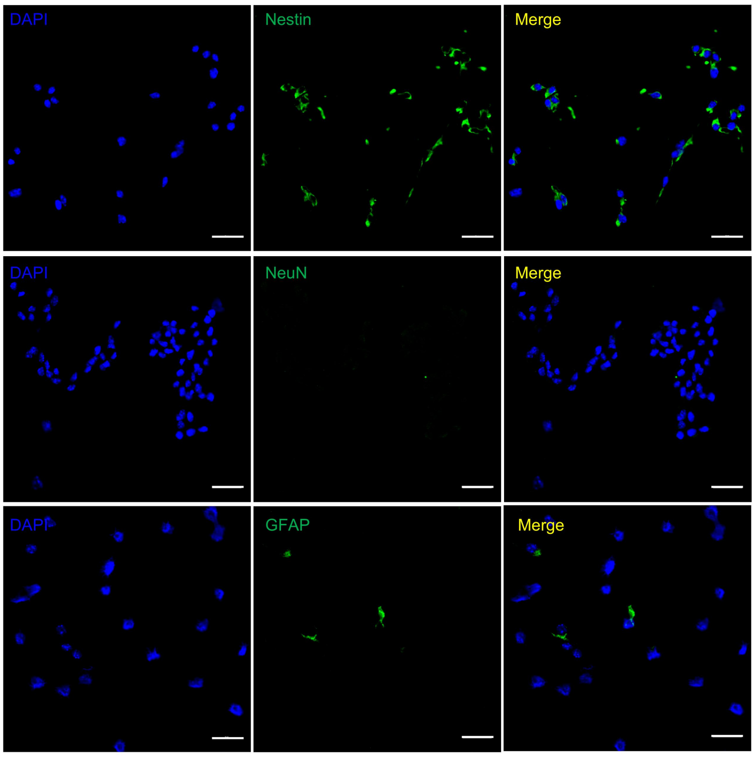

Figure S7. Immunofluorescent staining of Nestin, NeuN and GFAP to assess the purity of primary granule neuron cultures from Ctrl and cKO mice (related to Figure 5). Scale bar, 25 μm. DAPI was used for the counterstaining of nucleus.

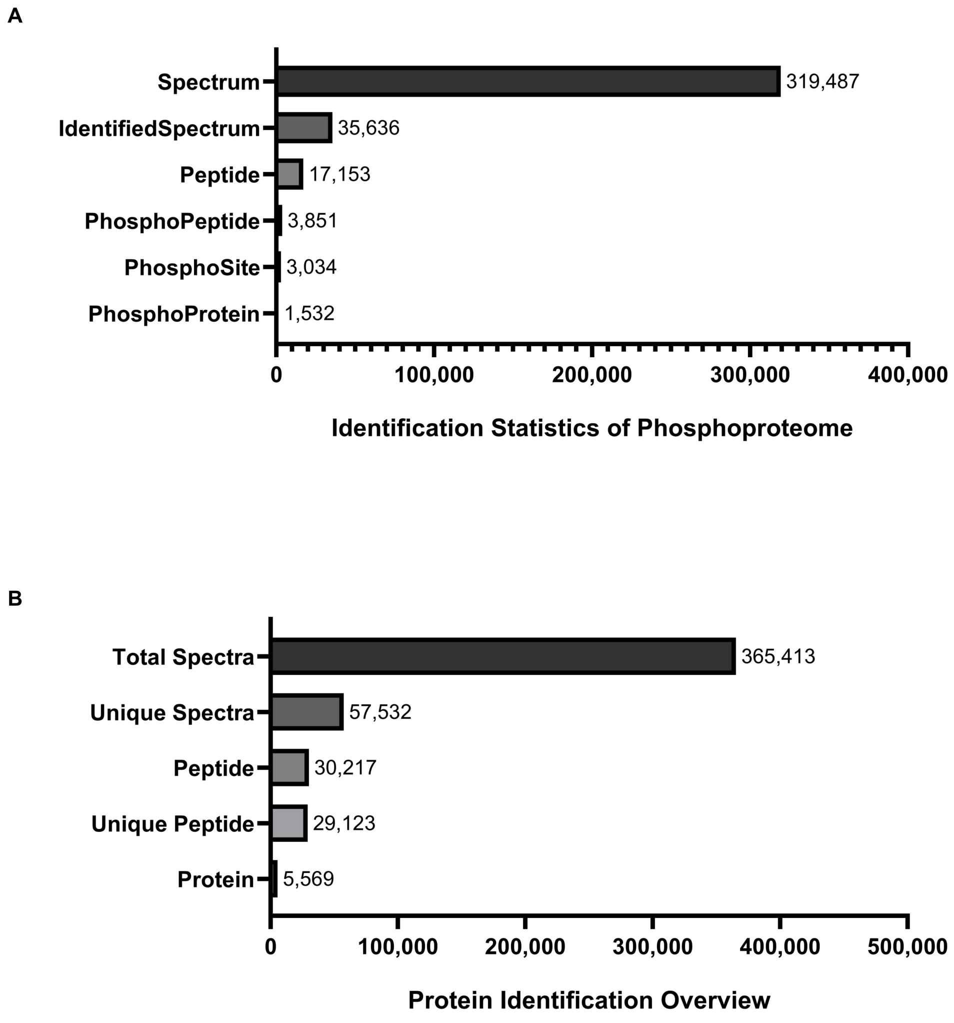

Figure S8. Summary of phosphoproteomic and proteomic profiling analysis of primary granule neurons from Ctrl and Atoh1-cKO mice (related to Figure 5).

(A) Protein identification overview of phosphoproteomic analysis. (B) Protein identification overview of proteomic analysis.

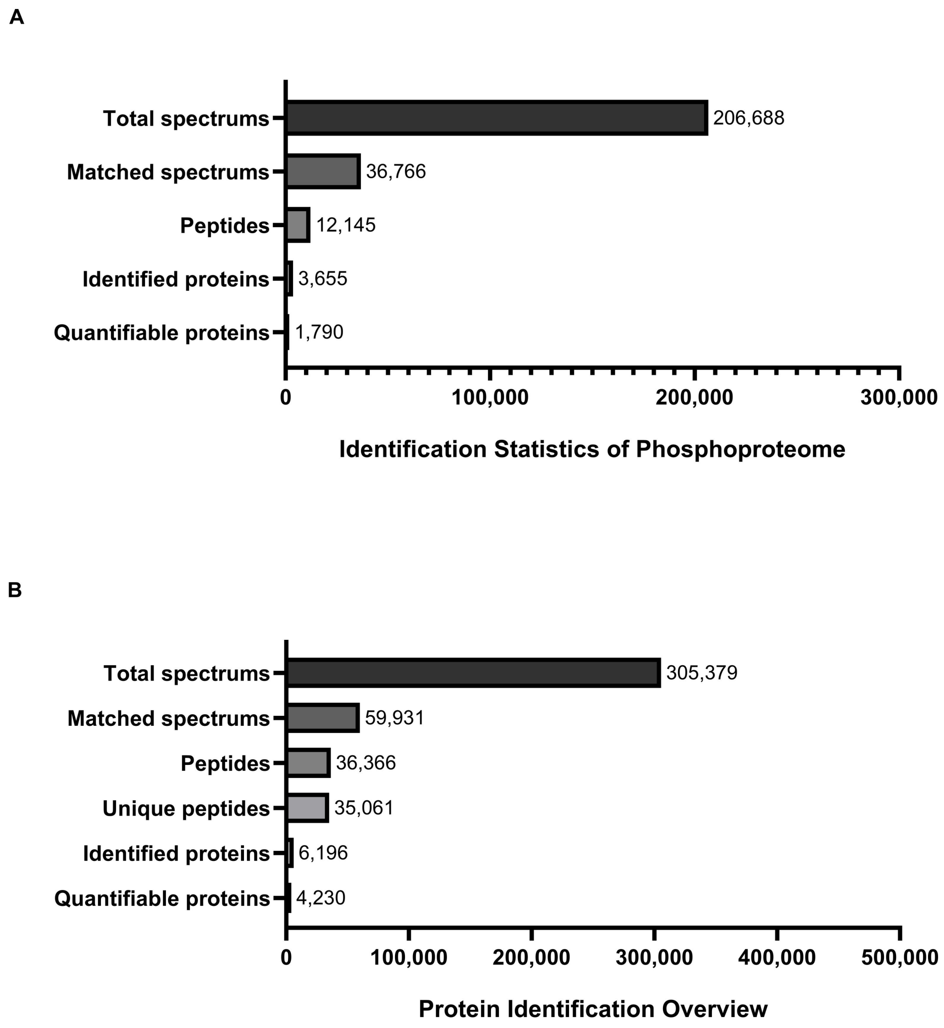

Figure S9. Summary of phosphoproteomic and proteomic profiling analysis of cerebellar cortices from Ctrl and Atoh1-cKO mice (related to Figure 6).

(A) Protein identification overview of phosphoproteomic analysis. (B) Protein identification overview of proteomic analysis.

Supplementary tables

Table S1. Related to Figure 5. List of proteins with increased or decreased phosphorylation levels in primary granule neurons from Atoh1-cKO mice.

Table S2. Related to Figure 5. List of differentially expressed proteins in primary granule neurons from Atoh1-cKO mice.

Table S3. Related to Figure 5. List of 29 candidate substrate proteins of PP2Acα in the primary granule neurons.

Table S4. Related to Figure 6. List of proteins with increased or decreased phosphorylation levels in cerebellar tissues from Atoh1-cKO mice.

Table S5. Related to Figure 6. List of differentially expressed proteins in cerebellar tissues from Atoh1-cKO mice.
